# Artificial intelligence in migraine: a narrative review on the diagnostic, prognostic, and therapeutic applications

**DOI:** 10.1093/oons/kvaf004

**Published:** 2026-04-29

**Authors:** Mahtasadat Yadollahi, Sahba Azadikhah Jahromi, Sanaz Bordbar, Kimia Kazemzadeh, Abbas Tafakhori

**Affiliations:** Network of Neurosurgery and Artificial Intelligence (NONAI), Universal Scientific Education and Research Network (USERN), Tehran, Iran; School of Pharmacology and Pharmaceutical Sciences, Isfahan University of Medical Sciences, Hezarjarib St., Isfahan 8174673461, Iran; Network of Neurosurgery and Artificial Intelligence (NONAI), Universal Scientific Education and Research Network (USERN), Tehran, Iran; School of Mechanical Engineering, Iran University of Science and Technology (IUST), Narmak St., Tehran 1684613114, Iran; Network of Neurosurgery and Artificial Intelligence (NONAI), Universal Scientific Education and Research Network (USERN), Tehran, Iran; Students' Scientific Research Center, Tehran University of Medical Sciences, Enghelab St., Tehran 1416753955, Iran; Network of Neurosurgery and Artificial Intelligence (NONAI), Universal Scientific Education and Research Network (USERN), Tehran, Iran; Iranian Center of Neurological Research, Neuroscience Institute, Imam Khomeini Hospital Complex, Tehran University of Medical Sciences, End of Keshavarz Blvd., Tehran 1419733141, Iran; Network of Neurosurgery and Artificial Intelligence (NONAI), Universal Scientific Education and Research Network (USERN), Tehran, Iran; Iranian Center of Neurological Research, Neuroscience Institute, Imam Khomeini Hospital Complex, Tehran University of Medical Sciences, End of Keshavarz Blvd., Tehran 1419733141, Iran; Department of Neurology, School of Medicine, Tehran University of Medical Sciences, Enghelab St., Tehran 1416753955, Iran

**Keywords:** artificial intelligence, deep learning, diagnosis, migraine, prognosis, treatment

## Abstract

Migraine is a complex neurological disorder characterized by recurrent and often debilitating headaches, impacting the quality of life for millions worldwide. Over the past decade, artificial intelligence (AI) has emerged as a transformative tool in the field of migraine research and clinical management. This narrative review provides a comprehensive overview of the diagnostic, prognostic, and therapeutic applications of AI in the context of migraine. In the diagnostic realm, AI-based algorithms demonstrate promising capabilities in accurately identifying migraine patterns and subtypes from diverse clinical data sources, including symptom profiles, imaging studies, and genetic markers. These advancements hold the potential for personalized diagnosis and improved patient stratification. Furthermore, AI-powered prognostic models offer the ability to predict migraine progression, anticipate treatment responses, and assess long-term outcomes, thereby aiding in tailored therapeutic decision-making and patient counseling. In therapeutic applications, AI-driven technologies are enabling innovative approaches, including personalized treatment regimens, optimized medication management, and the development of novel therapeutic targets based on big data analytics and predictive modeling. Overall, the integration of AI into the study and management of migraine represents a paradigm shift, holding tremendous promise for advancing precision medicine, enhancing clinical decision support, and ultimately improving the lives of individuals affected by this pervasive neurological condition. This review elucidates the current landscape and future prospects of AI in the multifaceted field of migraine management.

## Introduction

Migraine is characterized as one of the most frequent forms of headache by its recurrent and episodic nature, often accompanied by sensory disturbances and autonomic dysfunction [1]. The global prevalence of migraines encompasses approximately one billion individuals, with the majority lacking immediate and suitable medical attention [2]. A significant diagnostic delay of up to 12–17 years is commonly observed among patients, thereby heightening the risk of subsequent complications [3]. Currently, the diagnosis of migraines primarily relies on a thorough evaluation of the patient's history. Although it takes into account attack frequency and the presence of aura, this method falls short in addressing the phenotypic heterogeneity of migraine. Consequently, misdiagnoses are widespread, leading to high rates of misdiagnosis and inappropriate pharmacological interventions [4].

For the management of migraines, treatment options include both pharmacological and non-pharmacological approaches. Lifestyle modifications, including the identification and avoidance of triggers such as fasting, stress, sleep patterns, environmental factors, and hormonal fluctuations in women, are commonly recommended [5]. Pharmacological interventions encompass triptans and dihydroergotamine, with nonspecific pharmaceutical agents comprising anti-inflammatory medications, opioids, and sumatriptan/naproxen [6].

In recent years, researchers have devised algorithms for migraine with varying degrees of accuracy [1]. Artificial intelligence (AI) is the discipline encompassing the study and comprehension of the advancement of intelligent machines. Its utilization extends across the medical field, encompassing a broad class of computational techniques capable of performing tasks typically requiring human intelligence, including pattern recognition, predictive modeling, and decision support. Within the medical domain, AI has found applications in fields such as medical imaging, omics data interpretation, clinical decision-making, and robotics [7]. In light of the challenges associated with categorization, artificial neural networks (ANN) have been employed to enhance migraine classifications [8]. AI can be leveraged to investigate the mechanism of migraine further and identify distinct biomarkers and contributors to the disease [9]. AI has been utilized to create automated predictors aimed at tackling migraine. Tools have been developed to further enhance the diagnosis of the condition [10]. Machine learning (ML) can be implemented in the clinical management of migraine [11]. AI can be employed to explore diverse mechanisms of migraine, thereby proposing alternative medications or devising novel medication strategies for migraine [12].

This review aims to address the recent application of AI in the diagnosis and treatment of migraine. It reviews the existing literature in this regard and discusses the reliability of AI usage for migraine management.

## Artificial intelligence in migraine diagnosis

Early diagnosis and treatment of migraine have significant importance not only due to their influence on recommendations for the management of acute migraine, but also for informing long-term management strategies and optimizing clinical outcomes [13–16]. Diagnostic approaches for migraine often include clinical questionnaires, symptom assessment, and neuroimaging techniques. Studies have reported various reliable questionnaires for diagnosing migraine, including ID-migraine [17–19], MS-Q [20–22], HARDSHIP questionnaire [23–26]. The Migraine Disability Assessment (MIDAS) questionnaire is also reliable for assessing disability and severity, improving treatment decisions [27]. In addition, the visual aura rating scale can be used to diagnose migraine with aura [28, 29].

Regarding symptom-based assessment, the diagnosis of migraine relies on a patient's reported history and clinical examination. Common symptoms include unilateral or bilateral pain distribution, pulsatile quality, moderate or severe pain, and exacerbation with physical activity [30]. Moreover, the diagnosis of migraine may also be made based on associated symptoms like nausea or vomiting, photophobia, and phonophobia [31].

Furthermore, advanced neuroimaging techniques are commonly used to investigate migraine biomarkers, with MRI being particularly reliable for identifying microstructural brain impairments in migraineurs. Structural MRI assesses gray and white matter alterations, while functional changes can be observed through functional MRI (fMRI) [32, 33]. The blood-oxygen-level-dependent (BOLD) fMRI technique is widely used for measuring the blood-oxygen-level-dependent signals [34].

Moreover, data retrieved from the electroencephalogram (EEG) records have helped diagnose migraine in various studies [35]. However, the research in this area is still in its infancy; additionally, the specificity of EEG in diagnosing migraine is low, and more investigation is necessary to determine its diagnostic value. Despite limitations, EEG analysis contributes to understanding migraine pathophysiology and may aid in the development of new therapeutic approaches [36].

In the following section, a selected number of studies on the application and diagnostic accuracy of AI algorithms in identifying individuals with migraine are reviewed.

### Artificial intelligence in migraine diagnosis: Questionnaires and clinical symptoms

In the previous section, the traditional methods for diagnosing migraine disease in both non-clinical and clinical settings were mentioned. In this section, some studies on the application and diagnostic accuracy of AI algorithms in identifying individuals with migraine are reviewed.

*Kwon* et al. (2020) conducted a study examining the application of machine learning algorithms for migraine diagnosis across various headache categories, based on self-administered questionnaires. The researchers employed advanced machine learning approaches to classify headache disorders into five major groups: migraine, tension-type headache (TTH), trigeminal autonomic cephalalgia (TAC), epicranial, and thunderclap headaches. The machine learning models in this study include the stacked XGBoost classifier, Random Forest (RF), Support Vector Machine (SVM), and K-Nearest Neighbors (KNN). Each model employs a distinct strategy for pattern recognition and classification, resulting in varying performance outcomes. These classifications were based on data collected from 2162 participants, divided into two cohorts of training (n = 1286) and test (n = 876). Moreover, to mitigate overfitting, a least absolute shrinkage and selection operator (LASSO) was applied to select the most relevant features from the 128-dimensional questionnaire dataset. The LASSO is a regression analysis method used for feature selection and regularization, particularly in high-dimensional datasets. LASSO works by applying a penalty to the regression model, shrinking some coefficients to zero, thus eliminating irrelevant or redundant features. This results in a simpler, more interpretable model that avoids overfitting. The LASSO was used with a stratified tenfold cross-validation, and features that appeared in at least three out of ten folds were selected as stable features. Then, the models were trained using the stable features. These models have been utilized for classifying headache subtypes through the four layers of migraine vs. non-migraine, TTH vs. non-TTH, TAC vs. non-TAC (epicranial and TCH), and epicranial vs. TCH. The results of the classifier performance for two cohorts have shown that the XGBoost model using the selected features by the LASSO method has achieved an accuracy of 82% for the training cohort and 81% for the test cohort. The baseline accuracy (i.e. assigning all cases as the dominant subtype) for the training and test cohorts was 67% and 68%, respectively. Additionally, with a sensitivity of 88% and specificity of 95% for the test cohort, the XGBoost classifier showed the best performance in distinguishing migraine disorder from other headache subtypes. The XGBoost classifier is an ensemble method that constructs a series of decision trees, optimizing performance by learning from previous mistakes. Furthermore, a comparison was made between the proposed classifier and other algorithms, such as RF, SVM, and KNN, with chosen features by the LASSO method, in which the results showed that the XGBoost model outperforms other methods (accuracy: 0.8071, minimum sensitivity: 0.5273, and minimum specificity: 0.4561 in the test cohort). In addition, feature selection using the LASSO method was compared with other feature selection approaches, such as support vector machine recursive feature elimination (SVM-RFE) and minimum-redundancy maximum-relevancy (mRMR) using the XGBoost algorithm. This investigation demonstrated that the LASSO method outperforms other methods, achieving an accuracy of 0.8071, the lowest sensitivity of 0.5273, and the lowest specificity of 0.4561 in the test cohort. This study introduces a new method for diagnosing migraine using machine learning algorithms applied to a self-administered questionnaire. However, the study has some limitations. First, although the study encompasses a broad age range (11–90 years), it was conducted at a single center, which limits the external validity of the findings. Future research should apply data from multiple centers to the algorithms to assess generalizability. Although self-reported questionnaires offer valuable insights, the study might be affected by potential biases, such as recall and response bias. These biases may influence the model’s real-world applicability. Moreover, exploring more advanced deep learning techniques, like autoencoders, could enhance performance by better handling high-dimensional data and improving classification between headache subtypes. Furthermore, the exclusion of secondary headache disorders from this study’s model represents a key limitation, as clinicians must rule out secondary headaches (e.g. those caused by tumors or vascular issues) in the diagnostic process. Despite these challenges, the use of a stacked ensemble of XGBoost classifiers in migraine classification represents a significant advancement in the field of migraine classification. The study’s results are also superior to previous research that focused on fewer headache subtypes [37].

Furthermore, electronic health record (EHR) data (n = 6032), comprising both structured and unstructured datasets, have been utilized in a recent study conducted by *Riskin* et al. in 2023 to build an AI diagnostic model. This study has applied machine learning algorithms and natural language processing (NLP) to both unstructured data (physicians’ notes, also referred to as ‘Advanced real-world evidence (RWE)’) and structured data (medication lists and insurance claims, also referred to as "Traditional RWE"). These technologies help identify patterns and concepts, such as recognizing migraine-related symptoms, even when the data is ambiguous. NLP involves the development of systems that can understand, analyze, and generate natural language text or speech and is an essential component of human-computer interaction. In this research, both approaches have been employed to identify individuals with migraine as well as to extract six migraine-associated symptoms. To compare the approaches, two manual annotators reviewed all 6032 patient encounters, identifying and labeling key concepts such as headache type and severity. A reference standard was then created, which served as the basis for internal validation; recall, precision, and F1 scores were calculated by comparing the model’s outputs against this manually annotated reference dataset. The findings from the detection of migraine and headaches using both methodologies have shown that the Advanced RWE approach demonstrates superior accuracy across all metrics compared to the Traditional RWE technique. The following reflects the performance of Advanced RWE and Traditional RWE on each metric: Recall, 66.6% for migraine and 29.6% for headache in Traditional RWE, and 96.8% and 92%, respectively, in Advanced RWE. Furthermore, for six migraine symptoms, Advanced RWE achieved recall and F1 scores ranging from 79.3% to 96.6% and 80.7% to 95.6%, respectively, far surpassing those of Traditional RWE (0.0% to 17.9% recall; 0.0% to 28.9% F1). These findings demonstrate that the Advanced RWE technique outperforms the Traditional RWE method in all measures. Although Advanced RWE showed strong performance in this study, its generalizability remains uncertain due to the use of data from a tertiary care setting. Validation across diverse populations, including primary care, pediatric, and geriatric groups, is needed to assess broader clinical relevance. The study states that variability in language and patient populations may affect the model’s performance; however, the observed improvements are unlikely to be attributable solely to these factors. Future studies should aim to assess the model’s generalizability and clinical applicability in a broader range of settings and patient groups, particularly given its potential to enhance migraine diagnosis and treatment management [38].

An innovative study by *Khan* et al. (2024), investigated the application of multiple machine learning algorithms, incorporating data augmentation techniques, to classify migraine headaches. This study employed data augmentation techniques to address class imbalances and enhance model robustness, thereby increasing the initial dataset of 400 patient records to 1447. Additionally, missing data were handled using appropriate imputation techniques, ensuring the dataset was complete and consistent. These data preprocessing steps, including SMOTE and imputation, enhanced the robustness of the models, improving their ability to generalize and accurately classify migraine subtypes. The patient records included 24 clinical attributes, and the implemented algorithms were support vector machine (SVM), K-nearest neighbors (KNN), random forest (RF), decision tree (DST), and deep neural networks (DNN). DNN, which achieved the highest accuracy in this study, excels at learning complex patterns through its layered architecture. Deep neural networks (DNNs) are a class of machine learning algorithms that are similar to artificial neural networks, aiming to mimic the information processing of the brain. DNNs have more than one hidden layer situated between the input and output layers. The dataset included various migraine classes, including with and without aura, familial hemiplegic migraine, sporadic hemiplegic migraine and basilar-type aura. The data was assigned to the algorithms before and after augmentation, and their performances were evaluated and compared. With augmentation, DNN reached 99.66% accuracy—an improvement of 2.16%—outperforming KNN (97.1%) and SVM (94.6%). The dataset was divided into training (n = 1157) and testing (n = 290) sets, representing an approximately 80/20 split, and model performance was evaluated on the held-out test set. Notably, the 2% enhancement in DNN’s performance, while a modest improvement, highlights the value of augmenting limited data to achieve more robust predictions in real-world scenarios where data may be scarce. Furthermore, the model's accuracy was validated by domain experts, with the DNN model achieving a 97% match in classifying migraine types compared to expert diagnoses, demonstrating its potential to assist clinicians in more accurately diagnosing migraines. While the proposed model demonstrates high accuracy using data augmentation, one important key factor must be considered. The study lacks external validation in real-world clinical settings or with independent datasets; thus, future work should focus on validating the model in multi-center or international cohorts, particularly to assess its performance across diverse patient demographics, including pediatric and geriatric populations. However, this research highlights the crucial role of AI and data augmentation in diagnosing migraines, particularly in settings with constrained medical resources [39].

*Kwon* et al. (2020), *Riskin* et al. (2023), and *Khan* et al. (2024) emphasize the potential of AI in migraine diagnosis; however, they employ different methods and data sources. *Kwon* et al. focused on self-administered questionnaires, using multiple machine learning models to classify headache subtypes. In contrast, *Riskin* et al. combined structured and unstructured data through NLP, which outperformed traditional structured-data approaches, highlighting the value of unstructured clinical information*.*

*Khan* et al. used data augmentation, applying multiple machine learning algorithms, including DNNs, to address class imbalance and improve robustness across diverse migraine subtypes. While all three studies underscore AI’s promise in improving migraine classification, *Kwon* et al. and *Khan* et al. used controlled patient datasets. In contrast, Riskin et al. explored real-world clinical data, offering broader clinical applicability but potentially more variability. To conclude, these studies leverage a variety of approaches, from questionnaire-based models to EHR-driven and data-augmented strategies, each advancing migraine diagnosis with varying trade-offs between accuracy, generalizability, and real-world relevance.

### Artificial intelligence in migraine diagnosis: Neuroimaging data

In a study by *Chong* et al. (2017), the researchers utilized resting-state functional connectivity (RS-FC) patterns derived from MRI data to develop a machine learning model for differentiating between migraine patients and healthy controls. To assess the diagnostic accuracy between 58 individuals with migraine and 50 healthy controls, a brain classification algorithm was employed, incorporating input features from 33 regions of interest (ROIs) known to be involved in pain processing. To identify these regions, existing studies were utilized to locate areas known to be involved in pain processing and that differ between migraine sufferers and healthy individuals in terms of structural or functional aspects. Standard preprocessing (SPM and DPARSF) was applied, including artifact removal, motion correction, normalization, and filtering of irrelevant signals. Regarding class imbalances, the authors addressed potential biases by setting equal prior probabilities for both the migraine and healthy control groups in the DQDA model. This approach helped mitigate the effect of any minor imbalances in class sizes. The study employed Diagonal Quadratic Discriminant Analysis (DQDA), a supervised machine learning algorithm, to classify patients with migraines from healthy controls. DQDA is a variant of Quadratic Discriminant Analysis (QDA) that assumes diagonal covariance matrices for each class, thereby simplifying the model and allowing for non-linear decision boundaries between classes. They used PCA to extract key components from RS-FC data and DQDA for classification, selecting the most relevant features through a stepwise procedure. Consequently, according to the outcomes of the classification analysis, it is clear that the 10-fold cross-validation approach has shown both the highest overall accuracy rate of 81.0% and the most superior accuracy rate of 86.1%. These values have been derived from just 6 out of 33 ROIs, demonstrating the significance of these regions in distinguishing between individuals with migraine and healthy individuals. However, the small sample size, lack of external validation, and focus on predefined ROIs limit the generalizability and granularity of the results. Larger studies with external validation are needed to address these limitations and improve the model's accuracy. Furthermore, the study focused exclusively on an adult population, comprising 58 migraine patients (mean age, 36.3 years; SD, 11.5 years) and 50 healthy controls (mean age, 35.9 years; SD, 11.0 years). It did not evaluate the performance of the AI model in pediatric or geriatric populations, who may present with different clinical characteristics and migraine patterns. This study has also shown that a longer duration of the disease correlates with increased classification accuracy, and vice versa. Individuals who have experienced migraine for more extended time durations have been classified more accurately (duration of disease >14 years, classification accuracy 96.7%, and duration of disease >15, classification accuracy 96.5%) than those with shorter time durations (duration of disease ≤14, classification accuracy 82.1%, and duration of disease ≤15, classification accuracy 82.7%) [40].

On the other hand, in a separate investigation regarding MRI data, modifications in cerebral blood flow (CBF) were assessed in individuals diagnosed with migraine with aura (MwA) in contrast to those without aura (MwoA) and healthy controls (HC). The research included a total of 88 participants who suffered from migraines, with 32 experiencing aura and 56 without aura, along with 44 healthy controls. In addition, another set of 30 patients with migraines was enrolled in the experiment, with 10 of them having auras. Furthermore, MRI data collection was performed on all subjects, and the data were processed using SPM12 and ASLtbx to generate CBF maps. Data processing also included a voxel-based comparison of normalized CBF between cohorts with MwA and MwoA. Machine learning techniques, including SVM, KNN, RF, naïve Bayes (NB), and linear discriminant analysis (LDA), were employed to distinguish between MwA and MwoA based on identified CBF features derived from MRI data. Findings demonstrated distinctive patterns of CBF changes in individuals within the MwA group when contrasted with those in the MwoA group and HC. The performance of the models was compared, and the SVM performed best when under 100 runs of five-fold cross-validation. SVM works by finding the optimal hyperplane that best separates data points into different categories, maximizing the margin between them. This margin is the distance between the hyperplane and the closest data points of each class, ensuring good generalization to new, unseen data. This SVM algorithm demonstrated promising discriminatory performance between the MwA and MwoA categories, achieving accuracies of 84.3% and 83.3%, and area under the curve (AUC) values of 0.872 and 0.860 in the training and testing sets, respectively, outperforming other machine learning approaches. In addition, variations in CBF were strongly associated with clinical symptoms, including the intensity of headaches, overall quality of life, and emotional state. However, there are some limitations to consider. The study lacked patients during early aura onset and did not analyze MwA subtypes. MRI was performed during the ictal phase, resulting in the loss of real-time data. Only six CBF features were used to avoid overfitting in a small sample. While the model showed acceptable robustness, further validation with larger, more diverse cohorts and detailed migraine subtype and progression data is needed to confirm its clinical utility. Despite these limitations, this study demonstrates the ability of machine learning-based methods to utilize CBF to distinguish between patients with migraine with and without aura, which contributes to the diagnostic process for migraine patients [41].

In another study conducted recently, the application of three advanced neural network models for analyzing EEG data was investigated to differentiate between epilepsy, migraine, and healthy subjects. This study introduces a novel spike encoding method combined with a partially observed ReSuMe STDP recurrent SNN structure, establishing a new online framework for categorizing EEG signals into the three aforementioned groups. Furthermore, a deep BiLSTM structure was used as a reference point for the study analysis. The third model was a NeuCube structure, a spiking neural network framework designed for analyzing intricate spatiotemporal data. EEG data recorded from 11 channels and 36 subjects, including those with epilepsy, migraine, and healthy controls, were assigned to the models. The pre-processing step included two filters, a bandpass filter (0.5–70 Hz) to focus on the frequencies of interest and a notch filter (50 Hz) to eliminate electrical noise. Importantly, no other techniques were used in this step, making the data manipulation clear. The results show that reservoir-SNN achieves an 85% accuracy and outperforms BiLSTM with 80% accuracy when both models have 1000 hidden units. Notably, the reservoir-SNN method reduces processing time by 54% compared to BiLSTM, making it well-suited for real-time applications. Although NeuCube achieves a 97% accuracy, which may be due to its ability to integrate spatial and temporal data, its processing time is nearly 49% longer than that of the proposed reservoir-SNN. Furthermore, several potential EEG channels were identified as biomarkers by the models, and the identification of these channels, especially F8 and C4, across various models significantly deepens our comprehension of epilepsy and migraine disorders. This study highlights the importance of neural networks in migraine classification. In other words, SNNs offer fast and energy-efficient EEG classification, which is especially useful in real-time and low-data scenarios; even though they often underperform compared to deep neural networks. In this study, a proposed RSNN using temporal features was compared to NeuCube and BiLSTM for classifying EEG signals from patients with suspected epilepsy or migraine. While RSNN had faster processing, it did not outperform NeuCube in terms of accuracy, highlighting the need to balance speed and performance based on application requirements [42].

*Akben* et al. (2012) also utilized EEG data to construct an AI-based model to differentiate migraine patients from healthy subjects. In this model, an artificial neural network (ANN) Classifier and machine learning (ML) algorithms have been employed to determine the most effective flash stimulation frequencies for detecting migraineurs. Following the identification of this specific frequency, the required time duration for diagnosing migraine conditions has been investigated using stimulation periods. Indeed, to achieve this particular period, an analysis of the spectral power has been employed and subsequently validated using the ANN. The results of the neural network classification analysis indicate that the specificity, sensitivity, and total classification accuracy scores achieved at a flash stimulation frequency of 4 Hz are 93.3%, 93.3%, and 93.3%, respectively. These values demonstrate the highest precision when compared to the corresponding scores obtained at frequencies of 2 Hz and 6 Hz. Furthermore, it has been shown that ML algorithms such as Multilayer Perceptron (MLP), Radial Basis Function (RBF), Learning Vector Quantization (LVQ), and Self-Organizing Map (SOM) exhibit optimal performance at a frequency of 4 Hz. At this frequency, these algorithms achieve accuracy levels of 93.3%, 83.3%, 80%, and 83.3%, respectively. Moreover, the neural network classification outcomes for different flash stimulation periods indicate that the model achieves the highest accuracy at 8 seconds (93.3%, 93.3%, and 93.3%), outperforming the accuracy achieved at other periods, such as 2 seconds, 4 seconds, 6 seconds, and 10 seconds. Therefore, the results of this study indicate that the optimal frequency and the necessary time duration for diagnosing migraine individuals are 4 Hz and 8 s, respectively [43].

Comparing these studies highlights the diverse approaches and data modalities employed for AI-based migraine diagnosis using neuroimaging data. While *Chong* et al. (2017) focused on RS-fMRI and identified key pain-processing regions for classification, the second study emphasized differences between migraine with aura and without aura using perfusion MRI, demonstrating how specific clinical subtypes can influence model performance. Both MRI-based studies achieved comparable accuracy levels, ranging from 81% to 86% for RS-FC and 83% to 84% for CBF features; however, the latter incorporated subtype differentiation and stronger clinical correlations. On the other hand, EEG-based studies, including those by Akben et al. (2012) and recent spiking neural network analyses, relied on electrophysiological signals rather than structural or perfusion imaging. EEG approaches often achieved higher accuracies (up to 97% for NeuCube and 93.3% for ANN at optimized flash frequencies), providing rapid, potentially real-time diagnostic capabilities. However, they typically involved smaller sample sizes and more controlled experimental conditions. Altogether, these findings highlight the potential of neuroimaging data for migraine diagnosis.

As described in the included studies, these AI models have clear implications for enhancing patient-centered outcomes in migraine care. By pre-screening patients using the most predictive features, these tools could give less-specialized providers the confidence to make accurate diagnoses sooner, enabling earlier intervention and more personalized treatment strategies. The automated, time-saving tools described can reduce diagnostic delays and minimize human error. The use of real-world evidence offers a foundation for more informed clinical and policy decisions, supporting individualized care plans and potentially reducing migraine-related disability and burden. Together, these approaches not only improve diagnostic accuracy and efficiency but also create pathways for better disease management, quality of life, and health equity, particularly when integrated into awareness campaigns, decision-support systems, and ongoing real-world validation efforts.

## Artificial intelligence in migraine prognosis

Migraine is a complex neurological condition shaped by a wide range of internal and external factors [44], which can lead to variability in headache severity and frequency over time. These changes may present as increasing or decreasing trends throughout the course of the disorder [45]. For example, factors such as medication overuse and depression have been associated with an increased risk of transitioning to chronic migraine [46, 47]. Additionally, various comorbid conditions are recognized as significant predictors for the development of chronic migraine. For instance, asthma and depression have been shown to act as risk factors for chronic migraine [48, 49]. Research also indicates that different subgroups of migraine patients may experience varying prognoses, reflecting the diverse nature of the disorder [50]. Understanding these distinctions is crucial for tailored management strategies. Several AI-driven approaches have been developed to improve migraine prognosis, analyzing patient data to identify patterns that may predict disease course and transition to chronic migraine. These approaches leverage environmental data, wearable technology, and symptom tracking to offer personalized risk assessments and treatment recommendations.

In a study conducted by *Katsuki* et al.*,* the influence of environmental factors on migraine was examined using data from a smartphone headache diary app. Two modeling approaches were employed: a Generalized Linear Mixed Model (GLMM) and a deep learning (DL) model that combined a Feedforward Neural Network (FNN) and eXtreme Gradient Boosting (XGB). The GLMM, using a negative binomial distribution, predicted hourly headache occurrences, while the DL model captured nonlinear temporal dependencies with lagged features. The study found significant associations between migraine occurrence and variations in atmospheric pressure, precipitation, and moisture content (p < 0.001). For the DL model, the RMSE was 10.2, and feature importance was assessed using Gini impurity reduction. This analysis identified key weather variables influencing predictions, particularly barometric pressure. The GLMM and DL-based models were validated on a separate dataset from December 2019 to November 2020. The models were applied to this temporal validation set without re-estimation or recalibration. The temporal validation results showed that the GLMM achieved an RMSE of 13.4 and R^2^ of 52.9%, while the DL model achieved an RMSE of 10.2 and R^2^ of 53.7%. However, the study had some limitations, including a skewed sample (mean age 34.0, 89.2% female), limiting generalizability, especially to pediatric and geriatric populations. Concerns about overfitting were noted, as the RMSE values were higher in the temporal validation, and individual-level calibration was not performed [51].

In an alternative study conducted by Siirtola et al., data retrieved from wearable sensors were utilized. In this study, sleep data from wrist-worn Empatica E4 devices were examined to detect precursory episodes associated with migraines. Quadratic Discriminant Analysis (QDA) and Linear Discriminant Analysis (LDA) were compared. LDA uses a linear decision boundary, while QDA uses quadratic boundaries, which better capture subject-specific variations. Feature selection was performed using sequential forward selection (SFS) with noise injection to prevent overfitting. For personal models, test samples from nights before migraines were carefully selected to avoid overfitting. The decision boundaries in QDA and LDA were based on class-specific Gaussian distributions (QDA) and a shared covariance matrix. Personalized models, using QDA, achieved an average balanced accuracy of 84.1%, outperforming LDA (70.2%) based on internal validation within each subject. For user-independent models, leave-one-out cross-validation (LOOCV) was used, where each subject’s data was tested against models trained on other subjects’ data. These models showed poorer performance (below 50%), highlighting the challenges of generalizing across subjects. No external validation was conducted, meaning the generalizability of these results to other populations remains uncertain. Additionally, the balanced accuracy metrics exhibited considerable variation among individual participants in detecting attacks, ranging from 60.4% to 95.2%. Consequently, this study posits that such technology may hold promise for facilitating early diagnoses of migraine episodes, which could be a primary factor in improving the overall prognosis of the disease. However, the small sample size (seven subjects) limits the generalizability of the results, and there was no external validation. Future research should aim to address these limitations by using larger, more diverse samples and conducting external validation. Additionally, the study focused on adult participants (30–60 years old) with no mention of pediatric or geriatric groups, suggesting the need for further research in these populations [52].

*Stubberud* et al. (2023) evaluated the application of machine learning in predicting migraine attacks using wearable technology*.* The study involved 18 migraine patients (with or without aura), who, over a four-week period, completed headache diaries and biofeedback sessions (measuring heart rate, peripheral skin temperature, and muscle tension) using an app. Several machine learning models were employed, including LR, SVM, RF, Gradient Boosting Machines (GBM), Adaptive Boosting (AdaBoost), and XGBoost. Feature selection was conducted using Recursive Feature Elimination with Cross-Validation (RFECV), which showed that the optimal number of features for the RF model was approximately 15, beyond which no significant performance gains were observed. Shapley Additive Explanations (SHAP) values were used to interpret feature importance, with key predictors including symptoms such as cravings, swelling, feeling cold, as well as physiological factors like heart rate, sleep duration, and the presence and severity of headaches. Missing data were handled through listwise deletion, and class imbalances (headache vs. no-headache days) were addressed by stratified data splitting. Among the employed models, the RF classifier emerged as the best-performing model, with an average cross-validated AUC of 0.68 (±0.01), a validation set AUC of 0.56, and an out-of-sample test set AUC of 0.62. The cross-validated AUC was obtained using three-fold cross-validation. RF performs well by handling heterogeneous feature types and capturing nonlinear interactions, making it suitable for this dataset. Its ability to capture complex interactions between features, including premonitory symptoms, physiological measurements, and self-reported diary data, played a crucial role in its superior performance. RF was followed by GBM, with similar AUCs (~0.68); however, RF showed slightly better performance in out-of-sample predictions (AUC of 0.62). The study highlights the potential of combining mobile health apps, wearable technology, and ML for forecasting migraine headaches, contributing to the prognosis of the patients. Despite its promising results, the study had several limitations, including a small sample size (18 participants), a lack of external validation, and a short data collection period. The study focused on adults aged 18–65, suggesting the need for future research to explore the applicability of these models to pediatric and geriatric populations [53].

While the studies reviewed demonstrate the potential of AI-driven models in predicting migraine attacks, they do not directly assess long-term migraine prognosis, such as the likelihood of progression to chronic migraine. Moreover, none of the included AI prediction studies reported improvements in patient-centred outcomes (e.g. HIT-6, MIDAS) following AI-guided interventions, underscoring a gap between short-term forecasting and measurable reductions in disability. In other words, although these studies provide valuable insights into migraine dynamics and help patients implement preventive strategies, they primarily address short-term symptom fluctuations rather than the evolution of the disorder over months or years.

Although the studies reviewed thus far primarily focus on attack prediction, Large Language Models (LLMs) offer an emerging AI-driven approach that could contribute to long-term migraine prognosis. In an innovative study, the effectiveness of large language models (LLMs), including ChatGPT 3.5 and 4.0, Google Bard, Meta Llama 2, and Anthropic Claude 2, in patient education was evaluated. Using clinical expertise and guidelines, neurologists designed 30 migraine-related queries that encompassed various aspects of migraine, from diagnosis to treatment options [4, 54, 55]. Each query was individually presented to the LLMs, and the performance of the LLMs was evaluated qualitatively by the panel using a 3-point scale (poor, borderline, good), with no formal cross-validation or test set evaluation. The Pearson χ2 test was used to assess statistical significance between the LLMs' ratings, but it does not reflect traditional model performance metrics such as accuracy or validation. Among the tested LLMs, with 96.7% appropriate responses, ChatGPT-4.0 demonstrated the highest accuracy, although the differences in the performances of LLMs were not statistically significant (Pearson χ2 test, *P* = 0.48). However, Google Bard had a higher ‘poor’ rating compared to the other LLMs. Additionally, none of the LLMs were able to have adequate performance when asked the question ‘In severe cases, are there surgical options available for treating migraine?’ This might be due to their limited ability to differentiate between migraine and secondary headaches, as well as the ongoing debate surrounding surgical treatments for migraine. These findings suggest that LLMs may function as assistive tools in providing relevant migraine-related information to patients, enhancing migraine prognosis, and supporting healthcare providers [56].

However, the use of LLMs for patient education and decision support comes with both promise and challenges. On the one hand, LLMs can provide quick, broad access to information, empowering patients with knowledge and supporting clinicians in decision-making processes [57]. Their ability to generate personalized, context-aware responses based on individual patient inputs offers the potential for tailored advice. This could be especially beneficial in the context of migraine, where symptoms and triggers vary significantly from one individual to another [58]. Furthermore, LLMs can help address information gaps in underserved regions, providing easily accessible resources to those without immediate access to specialists [59].

On the other hand, LLMs face significant limitations, particularly when applied to sensitive medical contexts like migraine care. One major challenge is their tendency to produce hallucinations—i.e. generating factually incorrect or misleading information that could misguide both patients and healthcare providers. This is especially problematic in medical settings, where accuracy is paramount [60]. LLMs also lack domain-specific training, meaning they may not fully understand complex clinical nuances, such as rare migraine subtypes, pharmacodynamics, or the latest evidence in headache management [61]. While general-purpose LLMs like ChatGPT are trained on a broad range of internet data, they are not explicitly trained on specialized medical datasets, which can result in gaps in their knowledge or misinterpretations of clinical guidelines [61]. Additionally, the use of LLMs in decision support raises concerns about liability—if an LLM provides incorrect guidance, it is unclear who would be held responsible for the resulting consequences [62].

These limitations highlight the need for caution when integrating LLMs into patient education and decision support. While they hold potential as assistive tools, they are far from a substitute for expert medical advice [61]. Further research should focus on improving their domain-specific accuracy and addressing issues such as hallucination, data integration, and validation, to ensure they are safe and effective for use in healthcare [62].

Together, these studies demonstrate how various AI algorithms can be utilized to enhance migraine prognosis; however, they differ in scope, model complexity, and clinical applicability. *Katsuki* et al. leveraged large-scale environmental data from smartphone diaries and applied both GLMM and DL models, effectively capturing population-level temporal patterns and nonlinear interactions. Nevertheless, their model did not offer much insight for individuals. In contrast, *Siirtola* et al. *and Stubberud* et al. focused on wearable sensor data to model patient-specific migraine episodes, with personalized models such as QDA and RF demonstrating higher predictive performance for individual participants, but the small sample sizes limited generalizability.

While wearable-based approaches emphasize short-term attack prediction with physiological and behavioral inputs, smartphone- and LLM-based methods provide broader insights, including patient education and population-level patterns, but often lack external validation or direct evaluation of long-term prognosis. Taken together, these studies highlight a clear trade-off: personalized models offer precision, while population-level models offer scalability. Balancing the two will be key to improving both short-term migraine management and long-term care.

The research efforts in migraine prognosis have also begun to translate into real-world applications. For instance, Migraine Buddy is a consumer-facing migraine tracking app developed by Healint. With millions of users, the app has become the data source for large-scale studies on real-world migraine burden and triggers. It allows users to log detailed information about their attacks, including timing, pain location and intensity, symptoms, suspected triggers, medications, and personal notes, while also collecting passive data, such as timestamps, location, and optional sleep/activity information from connected wearables. Healint promotes the app’s machine learning-powered features for identifying patterns and predicting migraines. Still, the details behind these models, including their structure and accuracy, haven’t been fully disclosed or peer-reviewed. It’s worth noting that most peer-reviewed papers using Migraine Buddy data focus on describing migraine burden rather than evaluating the app’s built-in prediction tools. Thus, while the platform offers promise, its predictive features should be interpreted cautiously unless supported by published, validated results [31, 63].

Furthermore, Eclara is a newer AI-marketed migraine and headache tracking app (appearing in app stores in 2024) that positions itself as an ‘AI-powered’ tracker to help users discover personalized triggers and predict headaches by combining user symptom logging with contextual datasets (weather, food, sleep, stress, menstrual/hormonal cycle inputs, and other lifestyle factors). Public descriptions in app store listings and developer materials highlight automated insights and prediction features. However, the app does not disclose its model architecture or provide peer-reviewed validation of its AI capabilities. Additionally, no peer-reviewed studies or independent evaluations related to Eclara appear in PubMed or the broader academic literature, indicating that there are no published accuracy metrics or clinical outcome data available. While Eclara represents a new generation of ‘AI-first’ migraine tracking apps, and may be helpful for user engagement and hypothesis generation, any claims about its predictive power or clinical value should be considered preliminary until supported by externally validated research.

## Artificial intelligence in migraine treatment

Migraine treatment can be divided into two main categories: pharmacological and nonpharmacological, which encompass therapeutic and preventive interventions. Proper treatments could maintain the quality of patients’ lives [31]. However, inadequate therapeutic interventions could lead to the development of chronic forms, along with significant socio-economic impacts [63]. Non-pharmacological treatment options are limited to lifestyle modifications to eliminate behaviors that could trigger migraine attacks and behavioral treatments [31, 64].

There are three primary drug categories for migraine acute treatment: specific (Triptans), non-specific (common analgesics such as acetaminophen and non-steroidal anti-inflammatory drugs (NSAIDs) [6]), and adjuvant treatments. In addition, several pharmacologic categories are utilized as preventive options, for instance, anti-convulsants, anti-depressants, and blood pressure medications [31]. While these traditional treatments improve quality of life for many patients, they come with limitations, such as contraindications and potential side effects. For example, Triptans are contraindicated in patients with uncontrolled hypertension, and coronary, cerebrovascular, and peripheral vascular disease due to their vasoconstrictive effects [65].

As a result, new therapeutic methods could expand treatment options, leading to a more precise adjustment of patients’ comorbidities and existing limitations with treatment methods. Furthermore, administering evaluation tools to assess the efficacy of treatment methods could pave the way for a more accurate understanding of therapeutic interventions, ultimately leading to the design of more effective treatment plans for patients. AI, due to its ability to handle large datasets [66], could be used in the evaluation of both traditional and new treatment methods. As a result, several studies have explored the use of AI algorithms in evaluating migraine treatment, some of which will be discussed in detail.

### Artificial intelligence in migraine treatment: Efficacy evaluation

Non-steroidal anti-inflammatory drugs (NSAIDs) are one of the most common pharmacological treatments for migraine; however, it might be challenging to predict whether the patient responds to such medications [67]. Hence, to evaluate the response to these drugs in managing migraine disease, one study was carried out by *Lu* et al. The study comprised 610 individuals diagnosed with migraines, categorized into two groups: non-responders and responders. The participants were asked to complete questionnaires that included demographic data, migraine characteristics, and psychiatric conditions. The reduction in headache intensity was assessed by comparing visual analogue scales (VAS) before and 2 hours after drug intake. Three multivariable LR models were developed from variables identified as significant in univariable analysis (p < 0.1). Model 1 included migraine-related factors (disease duration, headache severity, attack frequency); Model 2 contained psychiatric factors (anxiety, depression, sleep disturbance); and Model 3 combined all factors (C1). The probability estimates from these LR models were used as inputs for receiver operating characteristic (ROC) curve analysis. The retrieved data were split into two sets: training/validation (80%) and test (20%). On the training/validation set, a ten-fold cross-validation was used for parameter tuning. Three machine learning models — SVM, DT, and multilayer perceptron (MLP) — were selected for their balance of interpretability and performance. Each was trained using either the ‘risk factors’ set (migraine-related and psychiatric variables) or the ‘all factors’ set (including demographics). In addition, the influence of predictors on the effectiveness of the medicine was assessed using the Cochran–Mantel–Haenszel test. The results show that among the LR models, model 3 had the highest AUC (0.722). Moreover, the AUCs of the SVM, DT, and MPL models for risk factors in the test cohorts were 0.712, 0.741, and 0.715, respectively, while the AUCs for all factors were 0.744, 0.737, and 0.731, respectively. Additionally, no statistically significant performance difference was found between the LR and ML approaches. Although ML did not outperform LR, both approaches offer practical decision-support tools that potentially guide initial NSAID selection and avoid prolonged, ineffective therapy. These models could also enhance efficiency and improve patient quality of life by incorporating influential predictors such as headache frequency, intensity, disease duration, and psychiatric symptoms into the decision system. The results also demonstrated a link between the effectiveness of NSAIDs and several migraine attributes, such as the intensity and frequency of headaches, the duration of the condition, and psychological aspects like sleep disturbances, depression, and anxiety. In other words, the patients' reaction to NSAIDs decreased as the severity of their migraine and mental health issues increased. Despite its contributions, the study is subject to several limitations. These limitations included the real-world observational design without a control group, exclusion of patients taking prophylactic or antipsychotic medications, potential misclassification of migraine type due to the 12-week follow-up period, lack of medication history prior to the study, reliance on self-reported measures, and absence of assessment for the nocebo effect. External validation was not performed, which restricts generalizability. Only adults (≥18 years) were included; therefore, the applicability to pediatric or geriatric migraine populations remains unknown and warrants further investigation. Hence, these prognostic models might aid in enhancing the choice of drugs at the beginning of migraine therapy and preventing extended or ineffective treatment stages in patients. In addition, considering the critical factors in patients' responses to NSAIDs could help physicians optimize their treatment strategies and ultimately improve patients’ quality of life [68].

On the other hand, in a recent study, the response to NSAIDs in migraine treatment was evaluated using a machine learning model based on neuroimaging parameters, the percentage of amplitude (PerAF) from RS-fMRI, and gray matter volume (GMV) from voxel-based morphometry. After quality control and preprocessing (including spatial normalization to MNI space, smoothing, and motion/physiological noise regression), propensity score matching was used to divide 118 patients into responders and non-responders, and using multimodal MRI, six different features related to PerAF and GMV were extracted. A feature selection step was conducted using the LASSO and recursive feature elimination algorithms, and the extracted neuroimaging features were used to construct multiple ML models, including LR, SVM, RF, DT, K-nearest neighbor (KNN), multilayer perceptron, elastic network, light gradient boosting machine (Lightgbm), and extreme gradient boosting algorithms. Training used a stratified 7:3 train-test split with ten-fold cross-validation for hyperparameter tuning. To prevent overfitting, a 10-fold cross-validation was performed on the training data, and the OpenNEURO database was used as an external validation [69]. Consequently, the RF which was the model with the smallest predictive residuals was chosen and its performance was evaluated using various model metrics (ROCAUC, PRAUC, BACC, sensitivity, F1 score, PPV, and NPV); the mentioned metrics in the training data was 0.982, 0.983, 0.927, 0.976, 0.930, 0.889, and 0.973 and these amounts for the test data were 0.711, 0.648, 0.639, 0.667,0.649, 0.632, and 0.647. RF is an ensemble of decision trees, which is robust to overfitting and effective with high-dimensional data. A public dataset was used as an external validation for which the metrics were as follows: 0.631, 0.651, 0.611, 0.808, 0.656, 0.553, and 0.706. In addition, a correlation analysis was performed in the last step; as a result, it was found that the GMV of the left precuneus and the attack time in non-responders were positively correlated. The study suggests that multimodal neuroimaging features could serve as potential neural biomarkers for predicting NSAID efficacy in migraine. However, the study has some constraints: a small single-center sample, an external validation cohort drawn from non-migraine pain conditions, an inability to establish causality between neuroimaging changes and NSAID effects, reliance on self-reported drug use with potential non-compliance, and incomplete quantification of psychological factors despite efforts to balance anxiety/depression scores. These factors limit generalizability, and future directions include multicenter trials with larger migraine-specific external validation, the incorporation of computational psychophysiology to integrate psychological and physiological parameters, and the exploration of longitudinal neuroimaging to track treatment responses over time [70].

Furthermore, *Yang* et al. (2020) focused on one of the non-pharmacological methods. They conducted an investigation aimed at elucidating the significance of pre-treatment cerebral gray matter (GM) volume in predicting the efficacy of acupuncture in migraine patients without aura (MwoA). In addition to the traditional migraine interventions, acupuncture is frequently used in migraine patients; however, its effectiveness is controversial [71–73], increasing the need for investigation using state-of-the-art methods. For this purpose, a cohort of 41 individuals aged 18–65 years was randomly assigned to receive either real acupuncture or sham acupuncture. To monitor the frequency of migraine occurrences before and after the acupuncture intervention, participants maintained a headache diary. Furthermore, to assess longitudinal alterations in GM volume, MRI imaging conducted during the interictal phase and subsequent voxel-based morphometry analysis were employed. On the other hand, the LASSO method supplemented by a 10-fold cross-validation was used in the feature selection process. Additionally, to classify responders versus non-responders based on the selected features, a machine learning approach utilizing linear SVM was leveraged. Linear SVMs aim to find a hyperplane that maximizes the margin between classes. Unlike non-linear SVMs, linear SVMs use a straight decision boundary, are computationally simpler, and are suitable when data are approximately linearly separable. The results indicated that this algorithm exhibited promising performance, as evidenced by an accuracy of 83%, a sensitivity of 73%, a specificity of 85%, and an area under the ROC curve of 0.787, effectively distinguishing between responders and non-responders to acupuncture treatment among MwoA patients. The outcomes of acupuncture were found to associate directly with the initial GM volume in the parietal gyrus, middle frontal/inferior frontal gyrus, and cuneus, with responders exhibiting reduced GM volume in the frontal and cuneus areas in contrast to nonresponders, who showed increased GM volume in the parietal regions. In conclusion, this study demonstrates that pre-treatment GM volume could be leveraged to predict the outcome of acupuncture in migraine patients without aura, thus reducing unnecessary use of this therapeutic method and lessening medical costs. Nonetheless, several limitations exist. The sample size was small, follow-up data were limited due to dropouts, and only a single dataset was used, preventing external validation and confirmation of repeatability. Future studies should incorporate larger, multimodal imaging datasets and evaluate the predictive models across multiple populations to enhance clinical applicability. Additionally, no evaluation was performed for pediatric or geriatric populations, and further research is needed to generalize these findings beyond the adult population [74].

Furthermore, another study has been conducted to assess the efficacy of biofeedback as another commonly used therapeutic intervention for migraine. Biofeedback is a behavioral treatment that has been proven effective in decreasing migraine severity and frequency [64]. On the other hand, nitric oxide (NO) is a neurotransmitter that reacts with superoxide anions, leading to the decomposition of NO, a significant interaction in migraines [75]. In one study, the prognostic value of NO and superoxide dismutase (SOD), a metalloenzyme reducing the superoxide concentration, in treating migraines using biofeedback has been evaluated by ANN. The ANN called ARIANNA (artificial intelligence assistant for neural network analysis) has already been developed in other studies [76–78]. This model is a feed-forward neural network (FFNN) comprising an input layer, two hidden layers, and an output layer. The number of hidden units in each hidden layer was determined automatically by the model rather than being pre-specified. Input features included age, baseline MIDAS score, and oxidative stress biomarkers—SOD, NOx, and peroxides—while the model output was the post-treatment MIDAS score. All hidden and production units used a hyperbolic tangent activation function, and the network was trained online, updating weights sequentially until a stopping rule was reached. Pre- and post-treatment concentrations in blood samples were measured, and migraine severity, frequency, and disability were assessed using the Migraine Disability Assessment Score (MIDAS). Consequently, the correlation between the mentioned elements was evaluated, and the ANN was used to predict the post-treatment MIDAS. Clinically, biofeedback treatment led to a substantial reduction in migraine disability, with mean MIDAS scores decreasing from 37.0 ± 13.2 pre-treatment to 18.8 ± 8.6 post-treatment (p < 0.001). Pre- versus post-treatment MIDAS scores were highly correlated (R = 0.863, p < 0.001). Biochemical markers also showed significant modulation: SOD increased from 6.5 ± 1.0 to 8.0 ± 0.7 U/mL (p < 0.001), NOx increased from 23.7 ± 4.2 to 31.4 ± 3.0 μM (p < 0.001), and peroxides decreased from 145.8 ± 40.3 to 82.5 ± 21.3 U/mL (p < 0.001). Pre-treatment MIDAS correlated moderately with peroxide levels (R = 0.451, p = 0.046) but showed no significant association with NOx or SOD. The ARIANNA model demonstrated approximately 75% cumulative accuracy in predicting post-treatment MIDAS; 13 of 20 predictions were exact, with an additional two predictions within a 5-point tolerance. Variable importance analysis indicated that NOx was the most influential predictor, followed by peroxides, with age, SOD, and pre-treatment MIDAS contributing to a lesser degree. Notably, the relationship between NOx and post-treatment MIDAS was non-linear and dependent on peroxide levels within a restricted range (116–205 U/mL), highlighting the model’s ability to capture interactions not evident in simple correlations. As a result, the ANN proved to be a reliable tool for predicting therapeutic efficacy in this area and possesses the ability to identify potential responders to biofeedback treatment, thereby helping to provide personalized treatment strategies. However, the most crucial limitation of this study is the small sample size of 20 patients. Additionally, the model was not tested on independent datasets, making the generalizability uncertain, and the automatic determination of hidden units may lead to overfitting [79].

Clinical trials, a primary step of evaluating the efficacy of the medications, mostly have strict criteria for selecting participants [80]. Consequently, real-world data from clinical settings could help gain more accurate insights into the effectiveness and efficiency of clinical treatments [81]. As a result, a study conducted in 2022 created an AI-based model to measure the soft outcomes of migraine treatment and prevention using electronic health records (EHRs). In this study, NLP and ML algorithms were applied to extract features from unstructured data from EHRs and generate a composite migraine outcome score. The model's accuracy was evaluated in both scoring and data extraction, with success rates of 70% and 80%, respectively. The average F1 score of the model for extracting 11 features defined by two specialists was 92%. Furthermore, among the 2006 evaluated encounters, migraine outcome model scores based on automated feature extraction of data elements and scoring were an exact match and close match to the manually scored encounter for 77.2% and 82.2% of encounters, respectively. Features extracted from structured fields using SQL achieved an average F1 score of 32.1%. In contrast, automated extraction from unstructured data achieved an F1 score of 92%, indicating that clinician-authored notes more effectively capture the thought process behind diagnosis and ongoing management. These results highlight the potential for AI to inform migraine tracking outcomes based on real-world data. On the other hand, since structured data is often used for identifying clinical concepts, a comparison of automated extraction from unstructured data and manual extraction from structured data was provided in which the average F1 scores calculated were 92% and 32.1%, respectively, leading to the fact that unstructured data could be a more valuable source for determining migraine outcome. These findings collectively demonstrate that this model can be utilized in clinical decision-making by providing healthcare providers with a summary of patient progress. However, several limitations of the study were noted. First, the model was developed using data from patients likely to have migraine, and its performance in populations without a formal diagnosis remains untested. Second, the dataset was derived from a single tertiary care center, which may limit generalizability to other clinical settings. Third, NLP alone was insufficient for accurate feature extraction; AI-based inference was required to achieve performance thresholds. As language patterns and documentation practices vary between institutions, similar results cannot be assumed for other technologies or healthcare settings [82].

Another AI-based study has been conducted by *Chartier* et al. to interpret pain sketches to predict the outcome of a migraine surgical intervention. Trigger-site deactivation surgery is a controversial treatment option for refractory or contraindicated migraine patients; thus, identifying the proper candidates would be crucial in this surgical intervention [83] in which pain sketches have proven to predict poor surgical outcomes [84]. Nonetheless, the process of extracting the required information from the sketches could be complex and in need of experienced professionals. In this study, an RF algorithm was trained to predict the surgical outcome in three categories: less than 20%, greater than 50%, and greater than 80% in migraine headache index (MHI) reduction, based on 24 features. For each category, the AI algorithm defined a certainty value between 0 and 1, and the prediction accuracy was compared with human predictions. The results showed that the AI algorithm’s accuracy was higher than that of human evaluators in all three categories, with the best accuracy for poor prognosis, defined as less than 20% reduction in MHI, at 94%. The algorithm also recognized diffuse pain, facial pain, and pain at the vertex as strong predictors for poor surgical outcomes. The predictive accuracy in the other categories was also good for typical/intermediate pain patterns that resulted in greater than 80% and greater than 50% improvement with 84% and 91% accuracy, respectively. All things considered, this algorithm could be a proper tool to help less experienced practitioners correlate pain patterns with surgical outcomes. Consequently, with the help of other methods, candidates for this therapeutic intervention would be appropriately recognized, and AI-based pain sketch analysis could serve as a preoperative decision-support tool, helping surgeons identify the proper candidates [85].

When comparing pharmacological and non-pharmacological approaches in predicting treatment response for migraine, distinct trends emerge. NSAID response prediction has been extensively studied using both clinical factors and neuroimaging-based machine learning models. Clinical-feature-based models, such as LR or SVM, trained on migraine characteristics and psychiatric factors, achieved moderate predictive accuracy, highlighting the importance of headache severity, frequency, and psychological comorbidities in treatment outcomes.

Neuroimaging-based approaches, incorporating gray matter volume and resting-state fMRI metrics, demonstrated slightly higher predictive performance in training cohorts but faced challenges in generalizability due to small sample sizes and external validation from non-migraine datasets. In contrast, non-pharmacological interventions, such as acupuncture and biofeedback, rely on neuroimaging and physiological features to predict response, with acupuncture prediction models achieving accuracies of up to 83% and associating outcomes with pre-treatment gray matter volumes in specific cortical regions. Overall, both approaches underscore the multifactorial nature of migraine and suggest that integrating clinical, psychological, and neuroimaging data could optimize personalized treatment strategies.

It is also worth mentioning that these studies, by predicting who is most likely to benefit from treatments such as NSAIDs, acupuncture, biofeedback, or even surgical interventions, can help patients avoid prolonged trial-and-error approaches and instead receive therapies tailored to their individual profiles. This means that patients could gain faster relief, experience fewer side effects from ineffective treatments, and see meaningful improvements in quality of life. Moreover, AI applied to real-world data, such as electronic health records and patient-generated information, enables continuous monitoring of treatment outcomes, allowing clinicians to adjust care strategies proactively. Ultimately, these innovations shift the focus from one-size-fits-all treatments toward precision medicine in migraine care, empowering both patients and providers to make better-informed decisions and reducing the overall burden of the disease.

### Artificial intelligence in migraine treatment: Drug design

Computational methods have led to a significant improvement in drug design, where ML and AI algorithms can be highly beneficial. One of the receptors that could be a possible target for drug design studies in migraine headaches is the δ opioid receptor (δOR). δOR selective agonists could act as pain-reducing agents in migraine [86]. In this area, AI algorithms can be used in conjunction with pharmacological and computational methods to predict the pharmacodynamic and pharmacokinetic properties of therapeutic molecules. They could also be utilized to predict drug-target affinity and interaction in cooperation with molecular docking and molecular dynamics (MD) simulations. They could also be integrated into docking-based investigations as a filter to determine which molecules would be the best candidates for further studies, including pharmacological research [87]. Regarding MD simulations, several advances have been made due to the administration of AI algorithms. MD simulations consist of multiple steps, some of which AI has been applied to [87]. For instance, we have the development of ML-based force fields [88] and geometry optimization using AI algorithms [89, 90]. Altogether, AI algorithms can lower the costs of drug discovery along with maintaining or increasing their efficiency and accuracy.

So far, what we have discussed regarding drug design has been about AI’s ability to contribute to computational drug discovery methods. However, one device has been designed that uses AI algorithms as a tool for optimizing treatment plans. This self-administered device, called Relivion®, utilizes peripheral nerve stimulation (PNS), a novel treatment method for patients who are refractory or contraindicated. Relivion® is designed to deliver electrical stimulation via its three adaptive output channels and is supported by a custom mobile application designed for logging the data and uploading it to the cloud database, the data that acts as a resource for its AI algorithms. In other words, based on this database, the algorithms could provide treatment modifications, such as adjusting the stimulation dose. Altogether, the self-administration and dynamic modifications provided by the AI algorithms could lead to a dramatic improvement in the efficacy of migraine preventive and potentially abortive treatment [91].

**Figure 1:**
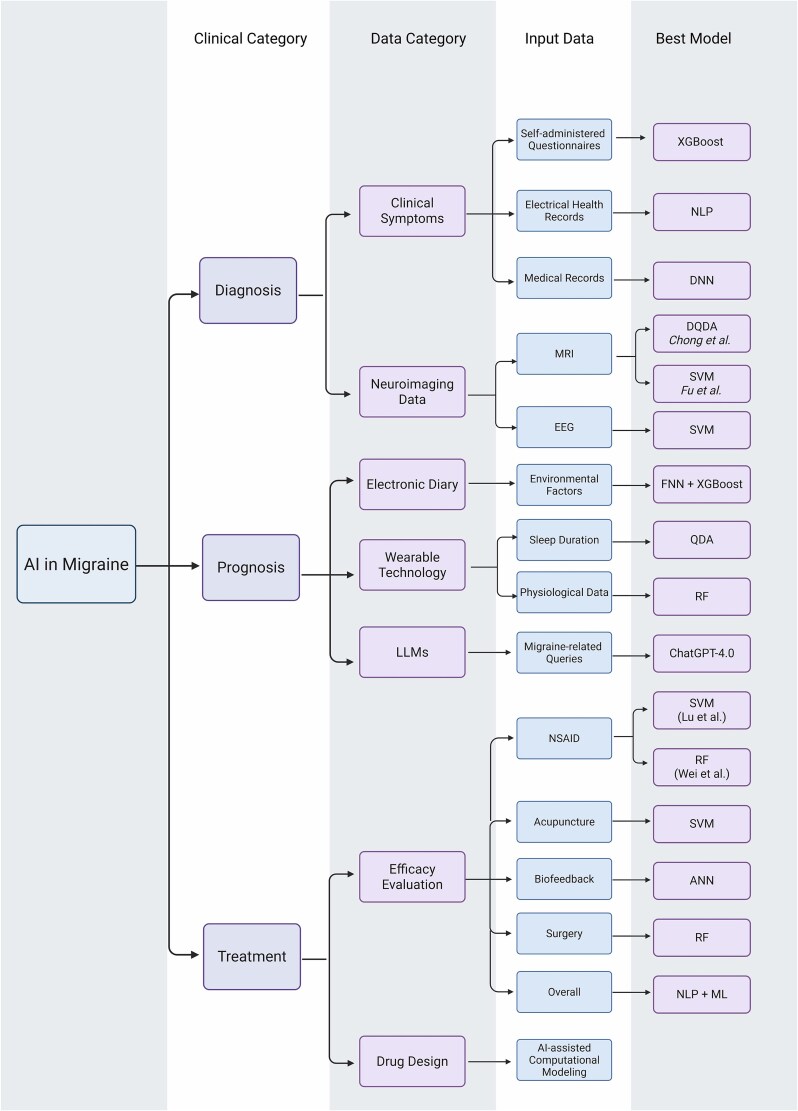
Summary of AI-related studies in migraine.

Check out Fig. 1 to find the summary of AI-related studies in migraine.

## Challenges and limitations

The use of AI-based methodologies has resulted in an improvement in the accuracy of diagnostic procedures, a reduction in needless referrals, a saving of time, a minimization of healthcare costs, and assistance to health facilities located in remote areas [92]. However, the implementation of algorithms in healthcare systems has caused several challenging problems.

Firstly, some of the models for migraine diagnosis and prognosis rely on patient-reported data and can be incomplete or subject to recall bias. Additionally, migraine symptoms such as aura or pain intensity are highly subjective, making AI-driven predictions complex. In addition, many migraine subtypes have overlapping symptoms, further complicating the diagnosis of the conditions.

Moreover, Many AI models are trained on single-center datasets, often with limited demographic diversity, which could result in biased algorithms that fail to generalize across different patient populations. Furthermore, using AI algorithms in migraine often requires access to sensitive patient data, which comes with the possibility of illegal use of patients' private data, including personal information and medical records [93].

Additional practical obstacles to integrating AI algorithms into migraine treatment strategies include potential algorithmic bias across demographic and comorbidity subgroups, clinician distrust driven by opaque models and workflow disruptions, data governance and privacy hurdles (GDPR/HIPAA), and the technical work required for safe EHR integration. These limitations undermine generalizability and slow translation into routine practice unless explicitly addressed in study design and submission packages [39, 94].

Furthermore, regulatory readiness for AI in migraine care is advancing but remains mixed across jurisdictions. In the United States, the FDA has articulated a clear pathway for AI/ML-based software as a medical device (SaMD) that emphasizes a lifecycle approach (predetermined change-control plans), Good Machine Learning Practice (GMLP), prospective validation, and plans for ongoing performance monitoring and retraining; manufacturers are encouraged to engage in early pre-submission discussions with the agency [95, 96]. In Europe, the EU’s AI Act together with the Medical Device Regulation (MDR) creates overlapping obligations for AI intended for medical purposes—classifying such systems as high-risk and requiring documentation on dataset quality, human oversight, risk management and conformity assessment—so teams planning to deploy migraine decision-support tools must prepare for both clinical evidence and new AI-specific compliance steps [97].

Check out Table 1 to find a summary of the role of AI in migraine and its related challenges.

**Table 1:** Artificial Intelligence in Migraine.

Artificial Intelligence (AI) in Migraine	Key AI Concepts	Artificial Intelligence	Enable the analysis of complex datasets to uncover patterns and insights relevant to migraine research.
		**Machine learning**	Algorithms that learn patterns from patient data (e.g. questionnaires, clinical records) to classify migraine versus other headache types.
		**Stacked XGBoost Classifier**	An ensemble method that combines multiple XGBoost models in layers to accurately distinguish migraine from other headache subtypes using selected questionnaire features.
		**Random Forest (RF)**	An algorithm that builds multiple decision trees from migraine patient data and averages the predictions to improve reliability in headache classification.
		**Support Vector Machine (SVM)**	A classifier that identifies the best boundary to separate migraine from other headache disorders based on patient-reported features.
		**Linear SVM classification**	A classification algorithm creating a linear boundary; interpretable and computationally simpler for linearly separable data.
		**K-Nearest Neighbors (KNN)**	A method that classifies a patient’s headache type by comparing their questionnaire responses to the most similar cases in the dataset.
		**Deep Neural Networks (DNN)**	Layered neural networks that learn complex patterns in migraine patient data to improve the classification accuracy of migraine subtypes.
		**Natural language processing (NLP)**	AI techniques applied to unstructured clinical notes to extract migraine-related information such as headache type, frequency, and severity.
		**Decision tree (DST)**	DST builds a tree structure to make decisions based on splitting criteria
		**Diagonal Quadratic Discriminant Analysis (DQDA)**	A supervised ML classifier that assumes diagonal covariance matrices for each class. Used to distinguish migraine patients from healthy controls based on brain connectivity patterns.
		**Naïve Bayes (NB)**	A probabilistic classifier based on Bayes’ theorem assuming feature independence.
		**Linear Discriminant Analysis (LDA)**	A classifier that projects data onto a lower-dimensional space to maximize class separability.
		**Artificial Neural Network (ANN)**	Learns complex relationships in migraine imaging, EEG, or functional data to differentiate between different groups
		**Multilayer Perceptron (MLP)**	Feedforward network with input, hidden, and output layers
		**Radial Basis Function (RBF) Network**	Uses radial basis functions as hidden layer activations; outputs are weighted sums of RBF responses.
		**Learning Vector Quantization (LVQ)**	Learns representative prototypes for each class; classifies new data by the closest prototype in feature space.
		**Generalized Linear Mixed Model (GLMM)**	A statistical model for predicting outcomes with both fixed and random effects; handles repeated measures.
			
		**Deep Learning (DL)**	Neural network-based models capturing complex, nonlinear relationships in data.
		**Feedforward Neural Network (FNN)**	A type of DL model where information moves forward through layers to make predictions.
		**Logistic Regression (LR)**	Linear model predicting the probability of binary outcomes.
		**Multivariable LR models**	LR models including multiple predictors simultaneously to improve prediction accuracy.
		**Large Language Models (LLMs)**	AI models trained on text to generate human-like responses and assist in knowledge tasks.
		**Decision Tree (DT)**	An ML model using tree-like rules to classify data; interpretable and can handle nonlinear relationships.
	**Applications in Migraine**	**Diagnosis**	AI-powered algorithms enhance diagnostic accuracy by distinguishing between migraine patients and individuals with other headaches or healthy individuals.
		**Prognosis**	Predictive models predict migraine attacks, enabling personalized therapeutic planning.
		**Treatment**	AI aids in evaluating treatment efficacy, discovering novel therapeutic targets, and tailoring interventions based on individual patient profiles
	**Challenges**	**Data Quality**	It may be incomplete or affected by recall bias.
		**Symptom variability**	Migraine symptoms (e.g. aura, pain intensity) are subjective, complicating predictions.
	**Challenges**	**Clinical Complexity**	Many migraine subtypes share symptoms, making diagnosis difficult.
		**Generalizability**	Models trained on limited, non-diverse populations may be biased.
		**Privacy & Security**	The use of personal and medical information poses significant legal and ethical risks.

## Future direction and conclusion

In future directions, it is essential to develop and validate AI algorithms capable of integrating various data modalities, including genetic profiles, neuroimaging, electronic health records, and patient-reported outcomes [98]. This comprehensive approach has the potential to yield a more holistic understanding of migraine pathophysiology and enable personalized, data-driven management strategies. Also, the implementation of AI-based predictive models for early detection of migraine onset and impending attacks represents a critical area for future research [99]. Leveraging longitudinal data and advanced machine learning techniques, the aim is to identify premonitory signs and prodromal symptoms, thereby facilitating timely interventions and preemptive treatment strategies [100]. Conducting prospective trials to evaluate the real-world effectiveness and cost-effectiveness of AI-enabled migraine care pathways will be imperative for widespread adoption and integration into routine clinical practice [101]. Exploring the potential of AI in elucidating the underlying mechanisms of treatment response and resistance, as well as in optimizing drug discovery and development for novel migraine therapeutics, holds immense promise for shaping the future landscape of migraine management [102].

Furthermore, the integration of AI-driven decision support systems into clinical practice requires rigorous assessment of their usability, impact on workflow efficiency, and patient outcomes. Clinical adoption pathways in the migraine field follow a pragmatic pathway: define a narrow, high-value intended use (e.g. primary-care triage, predicting attacks, or predicting preventive-medication response), assemble representative and well-annotated datasets, demonstrate robust retrospective and external validation, and then move to prospective implementation studies that measure clinician behavior and patient outcomes rather than only model metrics. Published migraine AI reviews and recent work on predicting treatment response emphasize that the most credible route to clinical uptake is evidence that the tool changes decisions or outcomes in real workflows (for instance, improving diagnostic accuracy, shortening time to effective therapy, or reducing attack burden). Parallel work, EHR/FHIR integration, clinician training, and payer engagement for coverage or coverage-with-evidence models must proceed in conjunction with clinical validation to make real-world adoption feasible [99, 103].

Current AI models in migraine management have demonstrated strong diagnostic accuracy, often aligning closely with expert consensus. For instance, a neuro AI model showed a robust correlation with expert diagnoses of neurological conditions, including migraine, achieving a Pearson correlation of 0.79 (p < 0.001) [104]. Moreover, advancements in explainable AI (XAI) have further enhanced the interpretability of these models. XAI refers to a set of methods that make the decision-making process of complex AI models more transparent and understandable to humans [105]. The use of XAI techniques provides clinicians with brain activation maps that help in understanding AI-generated decisions by visually highlighting the regions of the brain that contributed to a particular diagnosis [106]. demonstrated strong validity, helping to identify hidden or undiagnosed migraine cases in primary care settings [22]. In Table 2, a comparative summary of key studies on AI in migraine management was provided.

**Table 2:** Comparative summary of key studies on AI in migraine management.

Study	Top Performing Model	Input Data	Sample Size	Validation Approach	Highest Accuracy/AUC
Kwon et al. (2020)	XGBoost	Questionnaires	2162 participants	10-fold cross-validation	Accuracy: 81% (test cohort)
Riskin et al. (2023)	NLP	HER	6032 encounters	Internal validation	Recall: 96.8% (migraine detection in Advanced RWE)
Khan et al. (2024)	DNN	Medical Records	1447 records	Data Augmentation + Internal cross-validation	Accuracy: 99.66%
Chong et al. (2017)	DQDA	Resting-state fMRI	108 participants	10-fold cross-validation	Accuracy: 86.1%
Fu et al. (2017)	SVM	MRI (CBF)	132 participants	5-fold cross-validation	Accuracy: 83.3% (test set)
Saeedinia et al. (2024)	Reservoir-SNN	EEG	36 participants	Not Reported	Accuracy: 85%
Akben et al. (2012)	ANN	EEG	30 participants	Not Reported	Accuracy: 93.3%
Katsuki et al. (2020)	Deep Learning (FNN + XGB)	Smartphone headache diary app	4375 users	Temporal validation on a separate dataset	RMSE: 10.2, R^2^: 53.7%
Siirtola et al. (2020)	QDA	Wearable sensors (Empatica E4)	7 subjects	Internal validation + LOOCV	Accuracy: 84.1%
Stubberud et al. (2023)	RF	Wearable technology (heart rate, skin temperature, muscle tension) and headache diaries	18 participants	3-fold cross-validation + Out-of-sample test set	AUC: 0.68
Li et al. (2023)	ChatGPT-4.0	Migraine-related queries	30 queries	Qualitative evaluation by neurologists	96.7% appropriate responses
Lu et al. (2020)	SVM	Questionnaires	610 participants	Internal split (train/test 4:1) + 10-fold cross-validation	AUC: 0.744
Wei et al. (2023)	RF	Neuroimaging features	118 participants	Stratified 7:3 train-test split +10-fold cross-validation + external validation	Test: ROCAUC: 0.711
Yang et al. (2020)	SVM	MRI (Pre-treatment GM volume)	41 participants	10-fold cross-validation	Accuracy: 83%
Ciancarelli et al. (2020)	ANN	Age, baseline MIDAS score, SOD, NOx, peroxides	20 participants	Not Reported	Cumulative accuracy: ~75%
Hindiyeh et al. (2022)	NLP	EHR data	2006 encounters	Comparison to a manually annotated reference standard	Exact match to the manually scored encounter: 77.2%, Close match to the manually scored encounter: 82.2%
Chartier et al. (2023)	RF	Pain sketche	131 pain drawings	Comparison with human evaluator predictions	Poor prognosis accuracy: 94%, Typical/Intermediate pain: 91%, MHI > 80% improvement: 84% accuracy

All aspects considered, AI is revolutionizing migraine management by addressing key challenges in diagnosis, prognosis, and treatment. AI-driven diagnostic tools enhance accuracy by analyzing diverse data sources, including patient-reported symptoms, neuroimaging, and genetic markers, enabling the personalized and timely identification of migraine subtypes. Prognostic models powered by machine learning predict disease progression and treatment responses, enabling the development of tailored therapeutic strategies. Additionally, AI is advancing migraine treatment through innovations such as personalized medication regimens, novel drug discovery, and the optimization of existing therapies. While challenges such as data privacy and equitable access remain, the transformative potential of AI in migraine care is undeniable. Generally, AI is not merely a tool but a catalyst for reimagining migraine care. Its ability to synthesize complex data into actionable insights offers hope for improved patient outcomes and enhanced quality of life for millions affected by this debilitating condition. As research progresses, the collaboration between AI developers, clinicians, and policymakers will be pivotal in unlocking the full potential of AI in migraine management.

## Supplementary Material

Review_history_for_OXFNSC-2024-008_kvaf004_R3_kvaf004

## Data Availability

None.
