# Supplementary material for: Artificial intelligence in migraine: a narrative review on the diagnostic, prognostic, and therapeutic applications
Source: Oxf Open Neurosci. 2026 Apr 29;5:kvaf004. doi: 10.1093/oons/kvaf004 (PMC13137334; doi:10.1093/oons/kvaf004)
Supplement: Review_history_for_OXFNSC-2024-008_kvaf004_R3_kvaf004 [file review_history_for_oxfnsc-2024-008_kvaf004_r3_kvaf004.zip › Review history for OXFNSC-2024-008_kvaf004.R3_kvaf004.odt]

Review history for OXFNSC-2024-008.R3

Round 1 reviews
Reviewer 1
Recommendation – Major Revision
Comments to the Author
Needs major rewrite Define and explain/ the differences in AI.
SEE WIKEPEDIA FOR HOW TO WRITE THIS 
The manuscript covers the application of artificial intelligence (AI) to the diagnosis, prognosis, and treatment of migraines, but several areas would benefit from deeper explanations and stronger organization.


1. The introduction provides a general background on migraine but references AI-based studies for epidemiological claims. This risks conflating the prevalence of migraine with the technical performance of AI algorithms. The authors should include more robust epidemiological references and clarify how migraine heterogeneity and diagnostic delay data are established. Additionally, the transition from standard migraine management to AI applications appears abrupt. It would benefit from a more explicit rationale connecting the complexities of migraine to the unique capabilities of AI. Authors should add a transitional paragraph showing how AI specifically addresses known clinical challenges of migraine, which would help unify the text.


2. The manuscript reviews studies on questionnaires, EHR-based classification, and neuroimaging analyses in the diagnostic domain. While the accuracy metrics reported are meaningful, there is often limited detail on how these algorithms were tuned, validated, or tested for overfitting. Many datasets are small or come from single centers, which may limit external validity. Especially for neuroimaging and EEG-based approaches, the text does not consistently describe how artifact removal and feature extraction were handled. Without these methodological details on procedures or how specific metrics such as “minimum sensitivity” were derived, it is difficult for readers to gauge the robustness of the findings. The examples lack detailed discussion on model training approaches, dataset sizes, or cross-validation schemes. Authors should explain methodological details that would help readers assess the reproducibility and reliability of the findings. In addition, tying diagnostic performance more explicitly to concrete clinical baselines and how these AI tools compare with neurologists’ diagnostic accuracy would emphasize their value.


3. Discussing prognostic models highlights work involving environmental triggers, wearables, and large language models. Nonetheless, the section often merges short-term headache prediction with longer-term migraine prognosis without differentiating their distinct clinical endpoints. While predicting imminent attacks may be valuable, it is not the same as anticipating chronic migraine development. Moreover, references to large language models and patient education do not convincingly show how these relate to predicting migraine evolution. Further rationale is needed if such tools are to be integrated into the clinical workflow for prognosis rather than merely supplying educational content. Future directions should include multicenter trials or larger datasets and integrate potentially relevant comorbidities more explicitly in the predictive models.


4. In discussing the prognostic value of AI, the paper focuses on short-term or attack-based prediction via wearable sensors or weather variables but does not clarify how this aligns with long-term prognosis, such as preventing or managing chronic migraine. This distinction is essential because “prognosis” typically implies a broader or longer disease course. Moreover, references to large language models and patient education do not convincingly show how these relate to predicting migraine evolution; further rationale is needed if such tools are to be integrated into the clinical workflow for prognosis rather than merely supplying educational content.


5. Regarding treatment, the review illustrates how AI can predict responses to NSAIDs, acupuncture, or surgical interventions. Yet, it would be beneficial to detail how these models might be routinely applied in clinical practice. The discussion around neuroimaging-based prediction of drug efficacy would benefit from a stronger emphasis on sample size calculations, overfitting safeguards, and prospective validation. The manuscript also does not convey whether any of these AI frameworks have been externally validated in diverse clinical settings or whether they consider significant covariates such as comorbid conditions, medication adherence, or demographic variability.  Similar considerations should be applied to device use, where explaining the underlying AI approach, real-time patient monitoring, and regulatory status would clarify how this technology might integrate into standard care. Additionally, the section on AI for drug design introduces in silico strategies like MD simulations and docking but lacks direct migraine-specific examples; elaborating on how these methods have concretely led to novel analgesics or improved candidate filtering for migraine research would strengthen the narrative.


6. The review briefly addresses challenges related to data privacy, potential biases, and healthcare system integration. This section would be more substantial if it connected general AI concerns to the unique features of migraine research, such as reliance on patient-reported headache diaries and the complexity of aura vs. non-aura subtypes. Similarly, while the future directions mention multimodal data integration and prospective trials, the text could better define the scope for near-term vs. long-term objectives such as bridging single-site pilot studies to larger scale validations, coordinating AI development with established headache guidelines and ensuring user-friendly model interpretability for both clinicians and patients. Concluding with a more balanced tone that acknowledges AI’s promise while underscoring the need for thorough validation and ethical considerations would ensure the review’s comprehensiveness and practical relevance.


7. Page 4, Line 46 – The statement referring to the origin of AI in 1956 requires a supporting reference. While 1956 is typically acknowledged as the year AI research began, if authors refer to the Dartmouth Conference, the manuscript should include specific citations related to foundational AI history references to substantiate that claim.


8. Page 5, Line 4 – The explanation that AI can be divided into “virtual” and “physical” approaches may need refinement or additional context. Electronic devices can be placed in either category; here, the authors should clarify how devices that bridge informatics (virtual) and physical components, such as the mentioned wearable tech, would be classified to avoid ambiguity in the definitions.


Reviewer 2
Recommendation – Major Revision
Comments to the Author:
The manuscript covers the application of artificial intelligence (AI) to the diagnosis, prognosis, and treatment of migraines, but several areas would benefit from deeper explanations and stronger organization.
1. The introduction provides a general background on migraine but references AI-based studies for epidemiological claims. This risks conflating the prevalence of migraine with the technical performance of AI algorithms. The authors should include more robust epidemiological references and clarify how migraine heterogeneity and diagnostic delay data are established. Additionally, the transition from standard migraine management to AI applications appears abrupt. It would benefit from a more explicit rationale connecting the complexities of migraine to the unique capabilities of AI. Authors should add a transitional paragraph showing how AI specifically addresses known clinical challenges of migraine, which would help unify the text.
2. The manuscript reviews studies on questionnaires, EHR-based classification, and neuroimaging analyses in the diagnostic domain. While the accuracy metrics reported are meaningful, there is often limited detail on how these algorithms were tuned, validated, or tested for overfitting. Many datasets are small or come from single centers, which may limit external validity. Especially for neuroimaging and EEG-based approaches, the text does not consistently describe how artifact removal and feature extraction were handled. Without these methodological details on procedures or how specific metrics such as “minimum sensitivity” were derived, it is difficult for readers to gauge the robustness of the findings. The examples lack detailed discussion on model training approaches, dataset sizes, or cross-validation schemes. Authors should explain methodological details that would help readers assess the reproducibility and reliability of the findings. In addition, tying diagnostic performance more explicitly to concrete clinical baselines and how these AI tools compare with neurologists’ diagnostic accuracy would emphasize their value.
3. Discussing prognostic models highlights work involving environmental triggers, wearables, and large language models. Nonetheless, the section often merges short-term headache prediction with longer-term migraine prognosis without differentiating their distinct clinical endpoints. While predicting imminent attacks may be valuable, it is not the same as anticipating chronic migraine development. Moreover, references to large language models and patient education do not convincingly show how these relate to predicting migraine evolution. Further rationale is needed if such tools are to be integrated into the clinical workflow for prognosis rather than merely supplying educational content. Future directions should include multicenter trials or larger datasets and integrate potentially relevant comorbidities more explicitly in the predictive models.
4. In discussing the prognostic value of AI, the paper focuses on short-term or attack-based prediction via wearable sensors or weather variables but does not clarify how this aligns with long-term prognosis, such as preventing or managing chronic migraine. This distinction is essential because “prognosis” typically implies a broader or longer disease course. Moreover, references to large language models and patient education do not convincingly show how these relate to predicting migraine evolution; further rationale is needed if such tools are to be integrated into the clinical workflow for prognosis rather than merely supplying educational content.
5. Regarding treatment, the review illustrates how AI can predict responses to NSAIDs, acupuncture, or surgical interventions. Yet, it would be beneficial to detail how these models might be routinely applied in clinical practice. The discussion around neuroimaging-based prediction of drug efficacy would benefit from a stronger emphasis on sample size calculations, overfitting safeguards, and prospective validation. The manuscript also does not convey whether any of these AI frameworks have been externally validated in diverse clinical settings or whether they consider significant covariates such as comorbid conditions, medication adherence, or demographic variability. Similar considerations should be applied to device use, where explaining the underlying AI approach, real-time patient monitoring, and regulatory status would clarify how this technology might integrate into standard care. Additionally, the section on AI for drug design introduces in silico strategies like MD simulations and docking but lacks direct migraine-specific examples; elaborating on how these methods have concretely led to novel analgesics or improved candidate filtering for migraine research would strengthen the narrative.
6. The review briefly addresses challenges related to data privacy, potential biases, and healthcare system integration. This section would be more substantial if it connected general AI concerns to the unique features of migraine research, such as reliance on patient-reported headache diaries and the complexity of aura vs. non-aura subtypes. Similarly, while the future directions mention multimodal data integration and prospective trials, the text could better define the scope for near-term vs. long-term objectives such as bridging single-site pilot studies to larger scale validations, coordinating AI development with established headache guidelines and ensuring user-friendly model interpretability for both clinicians and patients. Concluding with a more balanced tone that acknowledges AI’s promise while underscoring the need for thorough validation and ethical considerations would ensure the review’s comprehensiveness and practical relevance.
7. Page 4, Line 46 – The statement referring to the origin of AI in 1956 requires a supporting reference. While 1956 is typically acknowledged as the year AI research began, if authors refer to the Dartmouth Conference, the manuscript should include specific citations related to foundational AI history references to substantiate that claim.
8. Page 5, Line 4 – The explanation that AI can be divided into “virtual” and “physical” approaches may need refinement or additional context. Electronic devices can be placed in either category; here, the authors should clarify how devices that bridge informatics (virtual) and physical components, such as the mentioned wearable tech, would be classified to avoid ambiguity in the definitions.
Decision letter

Manuscript ID OXFNSC-2024-008 entitled "Artificial Intelligence in Migraine: A Narrative Review on the Diagnostic, Prognostic, and Therapeutic Applications" which you submitted to the Oxford Open Neuroscience, has been reviewed.  The comments of the reviewer(s) are included at the bottom of this letter.

Please note that this journal operates with transparent peer review. This means that if your submission is accepted for publication, the full peer review history of your article will publish online alongside your article. This includes reviewer comments, editor decision letters, and your author responses.

The reviewer(s) have recommended publication, but also suggest some major revisions to your manuscript.  Therefore, I invite you to respond to the reviewer(s)' comments and revise your manuscript within the next 60 days from today date.

To revise your manuscript, log into https://mc.manuscriptcentral.com/oxfnsc and enter your Author Centre, where you will find your manuscript title listed under "Manuscripts with Decisions."  Under "Actions," click on "Create a Revision."  Your manuscript number has been appended to denote a revision.

You may also click the below link to start the revision process (or continue the process if you have already started your revision) for your manuscript. If you use the below link you will not be required to login to ScholarOne Manuscripts.

*** PLEASE NOTE: This is a two-step process. After clicking on the link, you will be directed to a webpage to confirm. ***

https://mc.manuscriptcentral.com/oxfnsc?URL_MASK=615aaa6673a148a8b5de12277bc72fda

You will be unable to make your revisions on the originally submitted version of the manuscript.  Instead, revise your manuscript using a word processing program and save it on your computer.  Please also highlight the changes to your manuscript within the document by using the track changes mode in MS Word or by using bold or colored text.

Once the revised manuscript is prepared, you can upload it and submit it through your Author Centre.

When submitting your revised manuscript, you will be able to respond to the comments made by the reviewer(s) in the space provided.  You can use this space to document any changes you make to the original manuscript.  In order to expedite the processing of the revised manuscript, please be as specific as possible in your response to the reviewer(s).

IMPORTANT:  Your original files are available to you when you upload your revised manuscript.  Please delete any redundant files before completing the submission.

Because we are trying to facilitate timely publication of manuscripts submitted to the Oxford Open Neuroscience, your revised manuscript should be uploaded within the next 60 days from today date.  If it is not possible for you to submit your revision in a reasonable amount of time, we may have to consider your paper as a new submission.

Once again, thank you for submitting your manuscript to the Oxford Open Neuroscience and I look forward to receiving your revision.


Reviewer: 1

Comments to the Author
The manuscript covers the application of artificial intelligence (AI) to the diagnosis, prognosis, and treatment of migraines, but several areas would benefit from deeper explanations and stronger organization.


1. The introduction provides a general background on migraine but references AI-based studies for epidemiological claims. This risks conflating the prevalence of migraine with the technical performance of AI algorithms. The authors should include more robust epidemiological references and clarify how migraine heterogeneity and diagnostic delay data are established. Additionally, the transition from standard migraine management to AI applications appears abrupt. It would benefit from a more explicit rationale connecting the complexities of migraine to the unique capabilities of AI. Authors should add a transitional paragraph showing how AI specifically addresses known clinical challenges of migraine, which would help unify the text.


2. The manuscript reviews studies on questionnaires, EHR-based classification, and neuroimaging analyses in the diagnostic domain. While the accuracy metrics reported are meaningful, there is often limited detail on how these algorithms were tuned, validated, or tested for overfitting. Many datasets are small or come from single centers, which may limit external validity. Especially for neuroimaging and EEG-based approaches, the text does not consistently describe how artifact removal and feature extraction were handled. Without these methodological details on procedures or how specific metrics such as “minimum sensitivity” were derived, it is difficult for readers to gauge the robustness of the findings. The examples lack detailed discussion on model training approaches, dataset sizes, or cross-validation schemes. Authors should explain methodological details that would help readers assess the reproducibility and reliability of the findings. In addition, tying diagnostic performance more explicitly to concrete clinical baselines and how these AI tools compare with neurologists’ diagnostic accuracy would emphasize their value.


3. Discussing prognostic models highlights work involving environmental triggers, wearables, and large language models. Nonetheless, the section often merges short-term headache prediction with longer-term migraine prognosis without differentiating their distinct clinical endpoints. While predicting imminent attacks may be valuable, it is not the same as anticipating chronic migraine development. Moreover, references to large language models and patient education do not convincingly show how these relate to predicting migraine evolution. Further rationale is needed if such tools are to be integrated into the clinical workflow for prognosis rather than merely supplying educational content. Future directions should include multicenter trials or larger datasets and integrate potentially relevant comorbidities more explicitly in the predictive models.


4. In discussing the prognostic value of AI, the paper focuses on short-term or attack-based prediction via wearable sensors or weather variables but does not clarify how this aligns with long-term prognosis, such as preventing or managing chronic migraine. This distinction is essential because “prognosis” typically implies a broader or longer disease course. Moreover, references to large language models and patient education do not convincingly show how these relate to predicting migraine evolution; further rationale is needed if such tools are to be integrated into the clinical workflow for prognosis rather than merely supplying educational content.


5. Regarding treatment, the review illustrates how AI can predict responses to NSAIDs, acupuncture, or surgical interventions. Yet, it would be beneficial to detail how these models might be routinely applied in clinical practice. The discussion around neuroimaging-based prediction of drug efficacy would benefit from a stronger emphasis on sample size calculations, overfitting safeguards, and prospective validation. The manuscript also does not convey whether any of these AI frameworks have been externally validated in diverse clinical settings or whether they consider significant covariates such as comorbid conditions, medication adherence, or demographic variability.  Similar considerations should be applied to device use, where explaining the underlying AI approach, real-time patient monitoring, and regulatory status would clarify how this technology might integrate into standard care. Additionally, the section on AI for drug design introduces in silico strategies like MD simulations and docking but lacks direct migraine-specific examples; elaborating on how these methods have concretely led to novel analgesics or improved candidate filtering for migraine research would strengthen the narrative.


6. The review briefly addresses challenges related to data privacy, potential biases, and healthcare system integration. This section would be more substantial if it connected general AI concerns to the unique features of migraine research, such as reliance on patient-reported headache diaries and the complexity of aura vs. non-aura subtypes. Similarly, while the future directions mention multimodal data integration and prospective trials, the text could better define the scope for near-term vs. long-term objectives such as bridging single-site pilot studies to larger scale validations, coordinating AI development with established headache guidelines and ensuring user-friendly model interpretability for both clinicians and patients. Concluding with a more balanced tone that acknowledges AI’s promise while underscoring the need for thorough validation and ethical considerations would ensure the review’s comprehensiveness and practical relevance.


7. Page 4, Line 46 – The statement referring to the origin of AI in 1956 requires a supporting reference. While 1956 is typically acknowledged as the year AI research began, if authors refer to the Dartmouth Conference, the manuscript should include specific citations related to foundational AI history references to substantiate that claim.


8. Page 5, Line 4 – The explanation that AI can be divided into “virtual” and “physical” approaches may need refinement or additional context. Electronic devices can be placed in either category; here, the authors should clarify how devices that bridge informatics (virtual) and physical components, such as the mentioned wearable tech, would be classified to avoid ambiguity in the definitions.


Reviewer 2
This manuscript presents a comprehensive review of the role of artificial intelligence (AI) in migraine diagnosis, prognosis, and treatment. The topic is highly relevant, and the integration of AI in migraine research is an area of growing interest. However, the manuscript requires major revision to improve clarity, structure, and scientific rigor.  Improvements should focus on clarity, organization, critical analysis, and reducing redundancy.
The following concerns should be addressed:

Major Revision Points:
1. Lack of Critical Evaluation of Studies
The review primarily summarizes existing studies without critically assessing their strengths, limitations, and methodological rigor.
Recommendation: Provide comparative discussions on the reliability, sample sizes, validation methods, and reproducibility of AI models. Discuss potential biases (e.g., overfitting, small datasets) and generalizability of results.

2. Overuse of Long, Complex Sentences
The manuscript contains dense and convoluted sentences, making it difficult to follow key arguments.

3. Repetitive Statements
Some concepts, particularly those related to AI applications, are repeated across different sections (e.g., the role of AI in classification and diagnosis appears in multiple places).

4. Insufficient Discussion on AI Challenges and Biases
While AI is presented as transformative, the discussion lacks depth on key challenges:
Data privacy concerns
Ethical implications
Algorithmic bias in training models
Interpretability of AI-based decisions in clinical settings

5. Unclear Structure in Certain Sections
Some sections lack logical flow, particularly in the diagnostic and therapeutic applications:
The discussion jumps between different AI methodologies without a clear transition.
Some subheadings do not effectively categorize the information.

6. Lack of a Clear Definition of AI Techniques
The manuscript references machine learning, deep learning, artificial neural networks, and NLP without providing sufficient background.
Recommendation: Include a concise introductory section defining key AI concepts in the context of migraine research.

7. Citation Issues and Limited Discussion of Key References
Some citations appear to be randomly placed rather than directly supporting the statement.
The manuscript lists numerous studies without adequately discussing why they are significant.

8. Figures and Tables Missing
The manuscript does not include any figures or tables to illustrate AI-based models, study comparisons, or key findings.

9. Conclusion is Weak and Generic
The conclusion repeats information rather than synthesizing key takeaways.
Authors’ response
Response Letter
Dear Editorial Office,
On behalf of my coauthors, I would like to thank you and the honorable reviewers for the accurate review and precious comments on our work. We really appreciate all of the efforts and time that were put into this. We carefully read your comments and tried to address your comments. A point-by-point response letter and answers to the comments are included below. If any further changes are required, we are eager to know.
Reviewer 1:
The manuscript covers the application of artificial intelligence (AI) to the diagnosis, prognosis, and treatment of migraines, but several areas would benefit from deeper explanations and stronger organization.
Comment 1. The introduction provides a general background on migraine but references AI-based studies for epidemiological claims. This risks conflating the prevalence of migraine with the technical performance of AI algorithms. The authors should include more robust epidemiological references and clarify how migraine heterogeneity and diagnostic delay data are established. Additionally, the transition from standard migraine management to AI applications appears abrupt. It would benefit from a more explicit rationale connecting the complexities of migraine to the unique capabilities of AI. Authors should add a transitional paragraph showing how AI specifically addresses known clinical challenges of migraine, which would help unify the text.
Response: Many thanks for your valuable time and effort in reviewing our manuscript. Your comments really helped us to improve our manuscript. We rewrote the introduction part and added some paragraphs to address these issues. Changes are highlighted in the manuscript.
Comment 2. The manuscript reviews studies on questionnaires, EHR-based classification, and neuroimaging analyses in the diagnostic domain. While the accuracy metrics reported are meaningful, there is often limited detail on how these algorithms were tuned, validated, or tested for overfitting. Many datasets are small or come from single centers, which may limit external validity. Especially for neuroimaging and EEG-based approaches, the text does not consistently describe how artifact removal and feature extraction were handled. Without these methodological details on procedures or how specific metrics such as “minimum sensitivity” were derived, it is difficult for readers to gauge the robustness of the findings. The examples lack detailed discussion on model training approaches, dataset sizes, or cross-validation schemes. Authors should explain methodological details that would help readers assess the reproducibility and reliability of the findings. In addition, tying diagnostic performance more explicitly to concrete clinical baselines and how these AI tools compare with neurologists’ diagnostic accuracy would emphasize their value.
Response: We really appreciate your comment. we tried to solve this issue by adding paragraphs and sentences discussing the method of mentioned studies. These sentences were highlighted in the manuscript.
Comment 3 & 4. Discussing prognostic models highlights work involving environmental triggers, wearables, and large language models. Nonetheless, the section often merges short-term headache prediction with longer-term migraine prognosis without differentiating their distinct clinical endpoints. While predicting imminent attacks may be valuable, it is not the same as anticipating chronic migraine development. Moreover, references to large language models and patient education do not convincingly show how these relate to predicting migraine evolution. Further rationale is needed if such tools are to be integrated into the clinical workflow for prognosis rather than merely supplying educational content. Future directions should include multicenter trials or larger datasets and integrate potentially relevant comorbidities more explicitly in the predictive models.
In discussing the prognostic value of AI, the paper focuses on short-term or attack-based prediction via wearable sensors or weather variables but does not clarify how this aligns with long-term prognosis, such as preventing or managing chronic migraine. This distinction is essential because “prognosis” typically implies a broader or longer disease course. Moreover, references to large language models and patient education do not convincingly show how these relate to predicting migraine evolution; further rationale is needed if such tools are to be integrated into the clinical workflow for prognosis rather than merely supplying educational content.
Response: Many thanks for your comment. We tried to solve this issue by rewriting some parts of this heading and adding information to the paragraphs.
Comment 5. Regarding treatment, the review illustrates how AI can predict responses to NSAIDs, acupuncture, or surgical interventions. Yet, it would be beneficial to detail how these models might be routinely applied in clinical practice. The discussion around neuroimaging-based prediction of drug efficacy would benefit from a stronger emphasis on sample size calculations, overfitting safeguards, and prospective validation. The manuscript also does not convey whether any of these AI frameworks have been externally validated in diverse clinical settings or whether they consider significant covariates such as comorbid conditions, medication adherence, or demographic variability. Similar considerations should be applied to device use, where explaining the underlying AI approach, real-time patient monitoring, and regulatory status would clarify how this technology might integrate into standard care. Additionally, the section on AI for drug design introduces in silico strategies like MD simulations and docking but lacks direct migraine-specific examples; elaborating on how these methods have concretely led to novel analgesics or improved candidate filtering for migraine research would strengthen the narrative.
Response: We appreciate your comment which really helped us to enhance this heading. We added some paragraphs to this heading to solve this issue. Changes were highlighted in the manuscript.
Comment 6. The review briefly addresses challenges related to data privacy, potential biases, and healthcare system integration. This section would be more substantial if it connected general AI concerns to the unique features of migraine research, such as reliance on patient-reported headache diaries and the complexity of aura vs. non-aura subtypes. Similarly, while the future directions mention multimodal data integration and prospective trials, the text could better define the scope for near-term vs. long-term objectives such as bridging single-site pilot studies to larger scale validations, coordinating AI development with established headache guidelines and ensuring user-friendly model interpretability for both clinicians and patients. Concluding with a more balanced tone that acknowledges AI’s promise while underscoring the need for thorough validation and ethical considerations would ensure the review’s comprehensiveness and practical relevance.
Response: Many thanks for your comment. We added a paragraph to this heading to address this issue.
Comment 7. Page 4, Line 46 – The statement referring to the origin of AI in 1956 requires a supporting reference. While 1956 is typically acknowledged as the year AI research began, if authors refer to the Dartmouth Conference, the manuscript should include specific citations related to foundational AI history references to substantiate that claim.
Response: Many thanks for your comment. We rewrote that part to solve this issue.
Comment 8. Page 5, Line 4 – The explanation that AI can be divided into “virtual” and “physical” approaches may need refinement or additional context. Electronic devices can be placed in either category; here, the authors should clarify how devices that bridge informatics (virtual) and physical components, such as the mentioned wearable tech, would be classified to avoid ambiguity in the definitions.
Response: Thank you for your comment. We rewrote that part to solve this issue. Again, we really appreciate your time and effort in reviewing our manuscript. Please let us know if any comment was misunderstood so we can solve the issue.
Reviewer 2:
This manuscript presents a comprehensive review of the role of artificial intelligence (AI) in migraine diagnosis, prognosis, and treatment. The topic is highly relevant, and the integration of AI in migraine research is an area of growing interest. However, the manuscript requires major revision to improve clarity, structure, and scientific rigor. Improvements should focus on clarity, organization, critical analysis, and reducing redundancy. The following concerns should be addressed:
Comment 1. Lack of Critical Evaluation of Studies
The review primarily summarizes existing studies without critically assessing their strengths, limitations, and methodological rigor. Recommendation: Provide comparative discussions on the reliability, sample sizes, validation methods, and reproducibility of AI models. Discuss potential biases (e.g., overfitting, small datasets) and generalizability of results.
Response: Many thanks for your valuable time and effort in reviewing our manuscript. Your comments really helped us to improve our manuscript. We rewrote some of the mentioned parts and added some paragraphs to address these issues. Changes are highlighted in the manuscript.
2. Overuse of Long, Complex Sentences
The manuscript contains dense and convoluted sentences, making it difficult to follow key arguments.
Response: Thank you for your comment. We rewrote some sentences to enhance the clarity of our manuscript.
3. Repetitive Statements
Some concepts, particularly those related to AI applications, are repeated across different sections (e.g., the role of AI in classification and diagnosis appears in multiple places).
Response: We appreciate your comment. We rewrote some parts to address this issue.
4. Insufficient Discussion on AI Challenges and Biases
While AI is presented as transformative, the discussion lacks depth on key challenges:
Data privacy concerns
Ethical implications
Algorithmic bias in training models
Interpretability of AI-based decisions in clinical settings
Response: Many thanks for your comment. We added a paragraph to this heading to address this issue.
5. Unclear Structure in Certain Sections
Some sections lack logical flow, particularly in the diagnostic and therapeutic applications:
The discussion jumps between different AI methodologies without a clear transition.
Some subheadings do not effectively categorize the information.
Response: Thank you for your comment. We rewrote some sentences to enhance the clarity of our manuscript.
6. Lack of a Clear Definition of AI Techniques
The manuscript references machine learning, deep learning, artificial neural networks, and NLP without providing sufficient background.
Recommendation: Include a concise introductory section defining key AI concepts in the context of migraine research.
Response: Many thanks for your comment. we tried to address this issue in the introduction part and the new table.
7. Citation Issues and Limited Discussion of Key References
Some citations appear to be randomly placed rather than directly supporting the statement.
The manuscript lists numerous studies without adequately discussing why they are significant.
Response: We appreciate your comment which helped us to improve our manuscript. We tried to consider this issue in our manuscript.
8. Figures and Tables Missing
The manuscript does not include any figures or tables to illustrate AI-based models, study comparisons, or key findings.
Response: We appreciate your comment. we added a table to our manuscript.
9. Conclusion is Weak and Generic
The conclusion repeats information rather than synthesizing key takeaways.
Response: Thank you for your comment. We rewrote that conclusion to solve this issue. Again, we really appreciate your time and effort in reviewing our manuscript. Please let us know if any comment was misunderstood so we can solve the issue.
Round 2 reviews
Reviewer 1
Recommendation – Major Revision
Comments to author:
The manuscript does not provide an explanation of what the models are or how they differ from each other.
Although many studies are cited, it remains unclear what evidence supports the value of these models.
Reviewer 2
Recommendation – Minor revision
Comments to author:
The authors have significantly improved the manuscript by clarifying various methodological and conceptual aspects, the introduction, and distinguishing between short-term and long-term migraine outcomes.

1. While the updated version discusses cross-validation and feature selection, it does not clarify how missing data and imbalances, such as aura versus non-aura, were handled. A brief overview of strategies for unique model architectures would improve confidence in the reported accuracies. Additionally, including information on how AI decisions are informed would benefit readers in this context.

2. Although the authors have discussed short-term and long-term predictions, it remains unclear whether AI-driven interventions enhance patient-centered outcomes, including headache-related disability. Besides the adult population, individuals with migraines in pediatric and geriatric groups exhibit unique clinical characteristics. The text should specify whether any evaluated AI tools target these demographics or if further research is needed. Incorporating at least one real-world example or acknowledging the absence of such instances would strengthen the manuscript's practical implications.

Reviewer 3
Recommendation – Major Revision
Comments to author:
The authors have been responsive to both reviewers' comments and have revised the manuscript substantially. The introduction has been improved to clarify the connection between migraine complexity and AI’s clinical potential. Methodological details regarding AI models used for diagnosis, prognosis, and treatment have been expanded, particularly for neuroimaging and questionnaire-based studies. The authors also addressed distinctions between short- and long-term prognosis and clarified the role of large language models. Additionally, limitations such as single-center data and generalizability are now better acknowledged.

However, several concerns remain. While methodological descriptions have been added, there is still limited critical appraisal of study quality, including sample size limitations, external validation status, and clinical relevance. The review is largely descriptive and would benefit from comparative synthesis across studies. Additionally, Table 1 remains underdeveloped and does not function effectively as a summary of key findings or methods. There are no figures or schematic overviews that visually map AI methods to data types or clinical outcomes, which could enhance clarity and utility for readers. Although the language has improved, some long and redundant passages remain. Finally, the review could benefit from a more prominent discussion of regulatory readiness, clinical adoption pathways, and integration of AI into real-world clinical practice, especially in the context of device-based or decision-support systems. These additions would elevate the manuscript from a broad narrative to a more incisive and practically impactful review. 

Decision letter
Manuscript ID OXFNSC-2024-008.R1 entitled "Artificial Intelligence in Migraine: A Narrative Review on the Diagnostic, Prognostic, and Therapeutic Applications" which you submitted to the Oxford Open Neuroscience, has been reviewed.  The comments of the reviewer(s) are included at the bottom of this letter.

The reviewer(s) have recommended publication, but also suggest some major revisions to your manuscript.  Therefore, I invite you to respond to the reviewer(s)' comments and revise your manuscript taking into consideration all the concerns raised by the reviewers within the next 60 days from todays date.

Please note that this journal operates with transparent peer review. This means that if your submission is accepted for publication, the full peer review history of your article will publish online alongside your article. This includes reviewer comments, editor decision letters, and your author responses.

To revise your manuscript, log into https://mc.manuscriptcentral.com/oxfnsc and enter your Author Centre, where you will find your manuscript title listed under "Manuscripts with Decisions."  Under "Actions," click on "Create a Revision."  Your manuscript number has been appended to denote a revision.

You may also click the below link to start the revision process (or continue the process if you have already started your revision) for your manuscript. If you use the below link you will not be required to login to ScholarOne Manuscripts.

*** PLEASE NOTE: This is a two-step process. After clicking on the link, you will be directed to a webpage to confirm. ***

https://mc.manuscriptcentral.com/oxfnsc?URL_MASK=ffdda9e62a17428b8b3c30c04e8d9d4e

You will be unable to make your revisions on the originally submitted version of the manuscript.  Instead, revise your manuscript using a word processing program and save it on your computer.  Please also highlight the changes to your manuscript within the document by using the track changes mode in MS Word or by using bold or colored text.

Once the revised manuscript is prepared, you can upload it and submit it through your Author Centre.

When submitting your revised manuscript, you will be able to respond to the comments made by the reviewer(s) in the space provided.  You can use this space to document any changes you make to the original manuscript.  In order to expedite the processing of the revised manuscript, please be as specific as possible in your response to the reviewer(s).

IMPORTANT:  Your original files are available to you when you upload your revised manuscript.  Please delete any redundant files before completing the submission.

Because we are trying to facilitate timely publication of manuscripts submitted to the Oxford Open Neuroscience, your revised manuscript should be uploaded within the next 60 days from todays date.  If it is not possible for you to submit your revision in a reasonable amount of time, we may have to consider your paper as a new submission.

Once again, thank you for submitting your manuscript to the Oxford Open Neuroscience and I look forward to receiving your revision.

Reviewer: 1

Comments to the Author
The manuscript does not provide an explanation of what the models are or how they differ from each other.
Although many studies are cited, it remains unclear what evidence supports the value of these models.

Reviewer: 3

Comments to the Author
The authors have been responsive to both reviewers' comments and have revised the manuscript substantially. The introduction has been improved to clarify the connection between migraine complexity and AI’s clinical potential. Methodological details regarding AI models used for diagnosis, prognosis, and treatment have been expanded, particularly for neuroimaging and questionnaire-based studies. The authors also addressed distinctions between short- and long-term prognosis and clarified the role of large language models. Additionally, limitations such as single-center data and generalizability are now better acknowledged.

However, several concerns remain. While methodological descriptions have been added, there is still limited critical appraisal of study quality, including sample size limitations, external validation status, and clinical relevance. The review is largely descriptive and would benefit from comparative synthesis across studies. Additionally, Table 1 remains underdeveloped and does not function effectively as a summary of key findings or methods. There are no figures or schematic overviews that visually map AI methods to data types or clinical outcomes, which could enhance clarity and utility for readers. Although the language has improved, some long and redundant passages remain. Finally, the review could benefit from a more prominent discussion of regulatory readiness, clinical adoption pathways, and integration of AI into real-world clinical practice, especially in the context of device-based or decision-support systems. These additions would elevate the manuscript from a broad narrative to a more incisive and practically impactful review.

Reviewer: 2

Comments to the Author
The authors have significantly improved the manuscript by clarifying various methodological and conceptual aspects, the introduction, and distinguishing between short-term and long-term migraine outcomes.

1. While the updated version discusses cross-validation and feature selection, it does not clarify how missing data and imbalances, such as aura versus non-aura, were handled. A brief overview of strategies for unique model architectures would improve confidence in the reported accuracies. Additionally, including information on how AI decisions are informed would benefit readers in this context.

2. Although the authors have discussed short-term and long-term predictions, it remains unclear whether AI-driven interventions enhance patient-centered outcomes, including headache-related disability. Besides the adult population, individuals with migraines in pediatric and geriatric groups exhibit unique clinical characteristics. The text should specify whether any evaluated AI tools target these demographics or if further research is needed. Incorporating at least one real-world example or acknowledging the absence of such instances would strengthen the manuscript's practical implications.

Associate Editor
Comments to the Author:
Please address the comments made by the reviewers.

Authors’ response
Response to Reviewers
Manuscript ID: OXFNSC-2024-008.R1
Title: Artificial Intelligence in Migraine: A Narrative Review on the Diagnostic, Prognostic, and Therapeutic Applications
Journal: Oxford Open Neuroscience

Date: 16-Aug-2025
Dear Editor,
We would like to express our sincere gratitude to you and the reviewers for the constructive comments provided on our manuscript entitled “Artificial Intelligence in Migraine: A Narrative Review on the Diagnostic, Prognostic, and Therapeutic Applications”. We greatly appreciate the time and effort that was taken to review our work. The feedback has been invaluable in helping us strengthen the quality and impact of the paper. In response to the reviewers' comments, we have carefully revised the manuscript, addressing each point raised. We believe that these revisions have significantly improved the clarity, depth, and quality of our work. Below, we provide a detailed point-by-point response to the reviewers' comments, outlining the changes made in the manuscript.
We hope that these revisions meet with your satisfaction, and we look forward to your feedback.
Thank you once again for your consideration.
Sincerely,

Authors

Response to Reviewer 1
The manuscript does not provide an explanation of what the models are or how they differ from each other.
Response: We appreciate this observation. To address this, we have added a clearer definition and description of the various AI models discussed in the manuscript, including their distinct characteristics and applications.
Action taken: For each study, the models with the best performance have been discussed in detail in each study, and the description of other models has been added to Table 1.
Location in manuscript: The updates are spread throughout the manuscript. Although many studies are cited, it remains unclear what evidence supports the value of these models.
Response: Thank you for pointing this out. We have included a comparative description of the performance of the models.
Action taken: In the studies that compared their findings with clinical evidence, we have now incorporated a more explicit discussion of these comparisons. In other cases, we critically assess the findings from the data mentioned in each study and outline the strengths and limitations of the models based on their performance in real-world applications. We also mentioned a more comparative perspective between the performance of different models included in the study.
Location in manuscript: The updates are spread throughout the manuscript.
Response to Reviewer 3
The authors have been responsive to both reviewers' comments and have revised the manuscript substantially. The introduction has been improved to clarify the connection between migraine complexity and AI’s clinical potential. Methodological details regarding AI models used for diagnosis, prognosis, and treatment have been expanded, particularly for neuroimaging and questionnaire-based studies. The authors also addressed distinctions between short- and longterm prognosis and clarified the role of large language models. Additionally, limitations such as single-center data and generalizability are now better acknowledged. However, several concerns remain. While methodological descriptions have been added, there is still limited critical appraisal of study quality, including sample size limitations, external validation status, and clinical relevance. The review is largely descriptive and would benefit from comparative synthesis across studies.
Response: We agree that a more critical appraisal is necessary. We have now included a comparative synthesis of study quality, specifically addressing sample size limitations, external validation status, and clinical relevance.
Action taken: Study limitations were included and discussed after all the included studies. Additionally, we have added one paragraph to each section comparing the included models (p.8, line 6-19, p.11 line 26-p.12 line 2, p.15 lines 12-26, p.22 line 25-p.23 line 2)
Location in manuscript: The updates are spread throughout the manuscript. Additionally, Table 1 remains underdeveloped and does not function effectively as a summary of key findings or methods.
Response: We have revised Table 1 to provide a more structured and informative summary.
Action taken: Table 1 has been completely revised and expanded. We have clarified the headings and added new columns for clarity.
Location in manuscript: Table 1 (p. 27-29).
There are no figures or schematic overviews that visually map AI methods to data types or
clinical outcomes, which could enhance clarity and utility for readers. Although the language has improved, some long and redundant passages remain.
Response: We acknowledge the need for visual clarity. We have added a new flowchart that maps AI methods to data types and their corresponding model performance. This addition should help readers better understand the relationship between the models and their
applications.
Action taken: A new figure (Fig. 1) has been added to the manuscript. The figure illustrates AI methods, data types, and model performances.
Location in manuscript: Fig. 1 (p. 30).
Finally, the review could benefit from a more prominent discussion of regulatory readiness, clinical adoption pathways, and integration of AI into real-world clinical practice, especially in the context of device-based or decision-support systems. These additions would elevate the manuscript from a broad narrative to a more incisive and practically impactful review.
Response: We acknowledge the need for a discussion devoted to regulatory readiness and transitioning AI algorithms into the real world. Thus, we have expanded the “Challenges and Limitations” and “Future Direction and Conclusion” sections.
Action taken: A new paragraph has been added to the “Challenges and Limitations” section discussing regulatory readiness, and one has been added to the “Future Direction and Conclusion” discussing clinical adoption pathways.
Location in manuscript: p. 24, lines 30–34, p. 25, lines 1–6, p. 25, lines 27–35, p. 26, lines 1–2.
Response to Reviewer 2
The authors have significantly improved the manuscript by clarifying various methodological and conceptual aspects, the introduction, and distinguishing between short-term and long-term migraine outcomes.
While the updated version discusses cross-validation and feature selection, it does not clarify how missing data and imbalances, such as aura versus non-aura, were handled. A brief overview of strategies for unique model architectures would improve confidence in the reported accuracies.
Response: We appreciate this important suggestion. We have clarified the handling of missing data and class imbalances, including strategies like imputation and stratified sampling for balancing the aura versus non-aura subgroups. We also provide a discussion of the unique model architectures employed in these studies.
Action taken: In the studies that included a description of how they handled missing data and class imbalance, we have included the description.
Although the authors have discussed short-term and long-term predictions, it remains unclear whether AI-driven interventions enhance patient-centered outcomes, including headache related disability.
Response: Thank you for pointing this out. We now explicitly discuss the impact of AI-driven interventions on patient-centered outcomes, particularly headache-related disability. We summarize recent studies on how AI models have been used to improve outcomes and quality of life for patients.
Action taken: Added a new paragraph to each section discussing how each study enhances patient-centered outcomes.
Location in manuscript: p. 12, lines 3-13, p. 14, lines 24-27, p. 23, lines 3-13.
Besides the adult population, individuals with migraines in pediatric and geriatric groups exhibit unique clinical characteristics. The text should specify whether any evaluated AI tools target these demographics or if further research is needed.
Response: We have updated the manuscript to specify that, while the majority of AI models have focused on adult populations, there is growing interest in pediatric and geriatric populations.
Action taken: For all the included study, the patients’ age distribution has been discussed.
Location in manuscript: The updates are spread throughout the manuscript.
Incorporating at least one real-world example or acknowledging the absence of such instances would strengthen the manuscript's practical implications.
Response: We agree that real-world examples would be valuable. We have now included two
real-world applications used for tracking migraine using AI algorithms.
Action taken: A discussion of two new AI-based applications, Migraine Buddy and Eclara, has been added to the “Artificial Intelligence in Migraine Prognosis” section
Location in manuscript: p. 15, line 27-p.17 line 15

Round 3 reviews
Reviewer: 2
Recommendation: Accept
Comments to the Author:
The revised manuscript, titled “Artificial Intelligence in Migraine: A Narrative Review on Diagnostic, Prognostic, and Therapeutic Applications,” provides a comprehensive and timely overview of the application of artificial intelligence (AI) and machine learning in migraine diagnosis, prognosis, and treatment. The authors have substantially improved the manuscript, notably through the inclusion of an expanded Table 1 that summarizes methodologies, a new Figure 1 providing a visual overview of AI applications in migraine, and more detailed discussions regarding study limitations, regulatory pathways, and future directions. These additions improve the clarity and relevance of the review.
Minor:
Line 28 - 'DNN shave more than one hidden layer.' Instead, it should be 'DNNs have more than one hidden layer.' Make sure to use the correct plural form when describing neural networks.

Reviewer: 3
Recommendation: Minor Revision
Comments to the Author:
The revised manuscript "Artificial Intelligence in Migraine: Clinical Applications and Future Perspectives" represents a substantial improvement over the previous version. The authors have made significant efforts to address the prior reviewer's concerns, and the current version presents a clearer, more structured, and informative overview of the field. The use of AI in migraine research is timely, and this review effectively summarizes diagnostic, prognostic, and therapeutic applications, while also acknowledging ongoing challenges in implementation.

Commendable Improvements:

The structure has been improved, with clearly delineated sections for diagnostic, prognostic, and treatment domains.

Explanations of AI models and algorithms (e.g., XGBoost, LASSO, SVM) have been added, making the review more accessible to a broader clinical audience.

The manuscript now provides more critical evaluations of studies, with attention to limitations such as small sample sizes and lack of external validation.

Challenges related to data privacy, bias, and regulatory constraints are now better elaborated.

The addition of Figure 1 and Table 1 enhances synthesis and readability.

Remaining Issues for Minor Revision:

Language clarity and editing. While the manuscript is generally well written, several minor grammatical issues and awkward phrasings remain. A thorough language editing pass (e.g., by a native speaker or professional editing service) is recommended to improve fluency and consistency.

Clarify model performance metrics. In several places, model performance is reported without specifying whether it refers to test set accuracy, cross-validation, or internal validation (e.g., Sections 2.1 and 3). This clarification is essential for readers to interpret the results reliably.

Comparative performance with clinicians. The review would benefit from a brief discussion of how current AI models compare to clinical judgment, even if limited data are available. Addressing this would place the reader’s expectations into context.

Large language models (LLMs). The section discussing LLMs (e.g., ChatGPT) is still somewhat superficial. Consider expanding the discussion to reflect both the promise and the challenges (e.g., hallucination, lack of domain-specific training) of using LLMs in patient education and decision support.

Long paragraphs in results discussion. Some paragraphs summarizing multiple studies (particularly in Sections 2.1, 3, and 4) are overly long and dense. Breaking these into smaller paragraphs would enhance readability.

Add a comparative summary table. A table summarizing the key studies discussed (e.g., model type, input data, sample size, validation approach, accuracy/AUC) would greatly help readers synthesize findings across studies and highlight trends or gaps in the field. 
Decision Letter:
Manuscript ID OXFNSC-2024-008.R2 entitled "Artificial Intelligence in Migraine: A Narrative Review on the Diagnostic, Prognostic, and Therapeutic Applications" which you submitted to the Oxford Open Neuroscience, has been reviewed.  The comments of the reviewer(s) are included at the bottom of this letter.

Please note that this journal operates with transparent peer review. This means that if your submission is accepted for publication, the full peer review history of your article will publish online alongside your article. This includes reviewer comments, editor decision letters, and your author responses.

The reviewer(s) have recommended publication, but also suggest some minor revisions to your manuscript.  Therefore, I invite you to respond to the reviewer(s)' comments and revise your manuscript within the next 30 days from todays date.

To revise your manuscript, log into https://mc.manuscriptcentral.com/oxfnsc and enter your Author Centre, where you will find your manuscript title listed under "Manuscripts with Decisions."  Under "Actions," click on "Create a Revision."  Your manuscript number has been appended to denote a revision.

You may also click the below link to start the revision process (or continue the process if you have already started your revision) for your manuscript. If you use the below link you will not be required to login to ScholarOne Manuscripts.

*** PLEASE NOTE: This is a two-step process. After clicking on the link, you will be directed to a webpage to confirm. ***

https://mc.manuscriptcentral.com/oxfnsc?URL_MASK=f1198a1e55fc47f38ced8e92c36d5fd3

You will be unable to make your revisions on the originally submitted version of the manuscript.  Instead, revise your manuscript using a word processing program and save it on your computer.  Please also highlight the changes to your manuscript within the document by using the track changes mode in MS Word or by using bold or colored text.

Once the revised manuscript is prepared, you can upload it and submit it through your Author Centre.

When submitting your revised manuscript, you will be able to respond to the comments made by the reviewer(s) in the space provided.  You can use this space to document any changes you make to the original manuscript.  In order to expedite the processing of the revised manuscript, please be as specific as possible in your response to the reviewer(s).

IMPORTANT:  Your original files are available to you when you upload your revised manuscript.  Please delete any redundant files before completing the submission.

Because we are trying to facilitate timely publication of manuscripts submitted to the Oxford Open Neuroscience, your revised manuscript should be uploaded within the next 30 days from todays date.  If it is not possible for you to submit your revision in a reasonable amount of time, we may have to consider your paper as a new submission.

Once again, thank you for submitting your manuscript to the Oxford Open Neuroscience and I look forward to receiving your revision.


Reviewer: 3

Comments to the Author
The revised manuscript "Artificial Intelligence in Migraine: Clinical Applications and Future Perspectives" represents a substantial improvement over the previous version. The authors have made significant efforts to address the prior reviewer's concerns, and the current version presents a clearer, more structured, and informative overview of the field. The use of AI in migraine research is timely, and this review effectively summarizes diagnostic, prognostic, and therapeutic applications, while also acknowledging ongoing challenges in implementation.

Commendable Improvements:

The structure has been improved, with clearly delineated sections for diagnostic, prognostic, and treatment domains.

Explanations of AI models and algorithms (e.g., XGBoost, LASSO, SVM) have been added, making the review more accessible to a broader clinical audience.

The manuscript now provides more critical evaluations of studies, with attention to limitations such as small sample sizes and lack of external validation.

Challenges related to data privacy, bias, and regulatory constraints are now better elaborated.

The addition of Figure 1 and Table 1 enhances synthesis and readability.

Remaining Issues for Minor Revision:

Language clarity and editing. While the manuscript is generally well written, several minor grammatical issues and awkward phrasings remain. A thorough language editing pass (e.g., by a native speaker or professional editing service) is recommended to improve fluency and consistency.

Clarify model performance metrics. In several places, model performance is reported without specifying whether it refers to test set accuracy, cross-validation, or internal validation (e.g., Sections 2.1 and 3). This clarification is essential for readers to interpret the results reliably.

Comparative performance with clinicians. The review would benefit from a brief discussion of how current AI models compare to clinical judgment, even if limited data are available. Addressing this would place the reader’s expectations into context.

Large language models (LLMs). The section discussing LLMs (e.g., ChatGPT) is still somewhat superficial. Consider expanding the discussion to reflect both the promise and the challenges (e.g., hallucination, lack of domain-specific training) of using LLMs in patient education and decision support.

Long paragraphs in results discussion. Some paragraphs summarizing multiple studies (particularly in Sections 2.1, 3, and 4) are overly long and dense. Breaking these into smaller paragraphs would enhance readability.

Add a comparative summary table. A table summarizing the key studies discussed (e.g., model type, input data, sample size, validation approach, accuracy/AUC) would greatly help readers synthesize findings across studies and highlight trends or gaps in the field.

Reviewer: 2

Comments to the Author
The revised manuscript, titled “Artificial Intelligence in Migraine: A Narrative Review on Diagnostic, Prognostic, and Therapeutic Applications,” provides a comprehensive and timely overview of the application of artificial intelligence (AI) and machine learning in migraine diagnosis, prognosis, and treatment. The authors have substantially improved the manuscript, notably through the inclusion of an expanded Table 1 that summarizes methodologies, a new Figure 1 providing a visual overview of AI applications in migraine, and more detailed discussions regarding study limitations, regulatory pathways, and future directions. These additions improve the clarity and relevance of the review.
Minor:
Line 28 - 'DNN shave more than one hidden layer.' Instead, it should be 'DNNs have more than one hidden layer.' Make sure to use the correct plural form when describing neural networks.

Associate Editor
Comments to the Author:
Please see the comments from the reviewers.
Authors’ Response:
Response to Reviewers
Manuscript ID: OXFNSC-2024-008.R2
Title: Artificial Intelligence in Migraine: A Narrative Review on the Diagnostic, Prognostic, and Therapeutic Applications
Journal: Oxford Open Neuroscience
Corresponding Author: Prof. Abbas Tafakhori
Date: 13-Oct-2025
Dear Editor,
We would like to express our sincere for your thoughtful and constructive feedback on our manuscript titled "Artificial Intelligence in Migraine: A Narrative Review on the Diagnostic, Prognostic, and Therapeutic Applications." We deeply appreciate the time and effort dedicated to reviewing our work. Your insights have been extremely valuable in enhancing the quality and impact of the paper.
In response to the reviewers' comments, we have made careful revisions to the manuscript, addressing each point raised. We believe these changes have substantially improved the clarity, depth, and overall quality of the work. Below, we provide a detailed, point-by-point response to the reviewers' comments, highlighting the adjustments made. We trust that these revisions will meet your expectations, and we look forward to receiving your further feedback. Thank you once again for your thoughtful consideration.
Sincerely,
Mahtasadat Yadollahi
On behalf of all authors

Response to Reviewer 3
The revised manuscript "Artificial Intelligence in Migraine: Clinical Applications and Future Perspectives" represents a substantial improvement over the previous version. The authors have made significant efforts to address the prior reviewer's concerns, and the current version presents a clearer, more structured, and informative overview of the field. The use of AI in migraine research is timely, and this review effectively summarizes diagnostic, prognostic, and therapeutic applications, while also acknowledging ongoing challenges in implementation.
Commendable Improvements: The structure has been improved, with clearly delineated sections for diagnostic, prognostic, and treatment domains. Explanations of AI models and algorithms (e.g., XGBoost, LASSO, SVM) have been added, making the review more accessible to a broader clinical audience. The manuscript now provides more critical evaluations of studies, with attention to limitations such as small sample sizes and lack of external validation. Challenges related to data privacy, bias, and regulatory constraints are now better elaborated. The addition of Figure 1 and Table 1 enhances synthesis and readability.
Remaining Issues for Minor Revision:
Language clarity and editing. While the manuscript is generally well written, several minor grammatical issues and awkward phrasings remain. A thorough language editing pass (e.g., by a native speaker or professional editing service) is recommended to improve fluency and consistency.
Response: Thank you for your helpful suggestion. We acknowledge the minor grammatical issues and awkward phrasings pointed out in the manuscript. To enhance the clarity and fluency, we have thoroughly reviewed the manuscript for language clarity and made necessary edits.
Action taken: The manuscript has undergone a comprehensive language editing process, including refining awkward phrasing, correcting grammatical errors, and ensuring consistency in language usage. We have also paid special attention to improving overall fluency to ensure the manuscript reads smoothly.
Location in manuscript: The updates are spread throughout the manuscript.
Clarify model performance metrics. In several places, model performance is reported without specifying whether it refers to test set accuracy, cross-validation, or internal validation (e.g., Sections 2.1 and 3). This clarification is essential for readers to interpret the results reliably.
Response: Thank you for pointing out the need for clarification regarding the model performance metrics. We agree that specifying the type of validation used is essential for readers to properly interpret the results. We have made sure to provide more detailed
explanations of the model performance metrics throughout the manuscript.
Action taken: We have explicitly clarified whether the reported model performance refers to test set accuracy, cross-validation, or internal validation in the relevant sections, particularly in Sections 2.1 and 3. Additionally, we have included specific details about the methodology used to calculate these metrics to improve transparency and help readers better interpret the results.
Location in manuscript: p. 5, line 35, p. 6, lines 3-4, p. 7, lines 33-35, p. 13, lines 27–33, p. 14, line 21, p. 15, lines 12-15,
Comparative performance with clinicians. The review would benefit from a brief discussion of how current AI models compare to clinical judgment, even if limited data are available. Addressing this would place the reader’s expectations into context.
Response: Thank you for your valuable suggestion. We agree that it would be beneficial to provide a comparison between the performance of AI models and clinical judgment. Including this comparison will help contextualize the findings and set realistic expectations for the reader regarding the practical utility of AI in migraine diagnosis and treatment.
Action taken: While data on this comparison is limited, we have added a brief discussion on the comparative performance of current AI models versus clinical judgment.
Location in manuscript: p. 27, lines 8-17
Large language models (LLMs). The section discussing LLMs (e.g., ChatGPT) is still somewhat superficial. Consider expanding the discussion to reflect both the promise and the challenges (e.g., hallucination, lack of domain-specific training) of using LLMs in patient education and decision support.
Response: Thank you for your insightful feedback. We agree that the section on Large Language Models (LLMs) can be expanded to better reflect both their potential and the challenges they face in practical applications. A more thorough discussion will provide a balanced view of the strengths and limitations of LLMs in the context of patient education and decision support.
Action taken: We have expanded the section on LLMs to provide a more comprehensive analysis. This includes a discussion of their promise in improving patient education and supporting decision-making, as well as the challenges associated with their use, such as
hallucinations, the need for domain-specific training, and the potential risks of inaccurate or incomplete information.
Location in manuscript: p. 15 line 25 – p. 16, line 13
Long paragraphs in results discussion. Some paragraphs summarizing multiple studies (particularly in Sections 2.1, 3, and 4) are overly long and dense. Breaking these into smaller paragraphs would enhance readability.
Response: Thank you for your valuable suggestion regarding the paragraph lengths. We agree that breaking down long and dense paragraphs into smaller ones will improve readability and make the manuscript more accessible to readers.
Action taken: In response to your feedback, we have split the long summarizing paragraphs in Sections 2.1, 3, and 4 into smaller, more focused paragraphs.
Location in manuscript: p. 8 lines 11-24, p. 16, lines 14-28, p. 23 line 27 – p. 24, line 16
Add a comparative summary table. A table summarizing the key studies discussed (e.g., model type, input data, sample size, validation approach, accuracy/AUC) would greatly help readers synthesize findings across studies and highlight trends or gaps in the field.
Response: Thank you for the excellent suggestion. We agree that a comparative summary table would be a valuable addition, as it would help readers quickly synthesize the key findings across the studies discussed and highlight trends or gaps in the field.
Action taken: We have added a comparative summary table to the manuscript. The table includes key information for each study, such as the model type, input data, sample size, validation approach, and performance metrics (e.g., accuracy or AUC).
Location in manuscript: Table 2 (p. 32 – 33)


Response to Reviewer 2
Comments to the Author The revised manuscript, titled “Artificial Intelligence in Migraine: A Narrative Review on Diagnostic, Prognostic, and Therapeutic Applications,” provides a comprehensive and timely overview of the application of artificial intelligence (AI) and machine learning in migraine diagnosis, prognosis, and treatment. The authors have substantially improved the manuscript, notably through the inclusion of an expanded Table 1 that summarizes methodologies, a new Figure 1 providing a visual overview of AI applications in migraine, and more detailed discussions regarding study limitations, regulatory pathways, and future directions. These additions improve the clarity and relevance of the review.
Minor: Line 28 - 'DNN shave more than one hidden layer.' Instead, it should be 'DNNs have more than one hidden layer.' Make sure to use the correct plural form when describing neural networks.
Response: Thank you for pointing that out. We agree that using the correct plural form is important for clarity and accuracy in the manuscript.
Action taken: The phrase "DNN shave more than one hidden layer" has been corrected to "DNNs have more than one hidden layer" to ensure proper grammar and consistency.
Location in manuscript: p. 7, line 28

Final Decision letter
It is a pleasure to accept your revised manuscript entitled "Artificial Intelligence in Migraine: A Narrative Review on the Diagnostic, Prognostic, and Therapeutic Applications" in its current form for publication in the Oxford Open Neuroscience. I have reviewed the changes and agree that the reviewers’ comments have been appropriately and satisfactorily addressed.

Please note that this journal operates with transparent peer review. This means that  the full peer review history of your article will publish online alongside your article. This includes reviewer comments, editor decision letters, and your author responses.

Next steps
You will receive an email from no-reply@scipris.com within roughly one week. This is your invitation to sign up for an account with SciPris, Oxford University Press’ author portal hosted by Aptara. You will need to create an account if you do not already hold one. Please register or log into your account and follow the online instructions which will guide you through signing your licence and paying the APC. The email and the portal have clearly signposted support options if you need any help during this process.

Please note that SciPris is a completely different system from ScholarOne, so your credentials to submit your manuscript here will not work there. Once you’ve created a SciPris account, you will be able to use it whenever you publish with Oxford Open Neuroscience or any OUP journal. Please note that OUP will only ever request payment for applicable fees be made via SciPris or to an OUP bank account. If you ever have concerns about the legitimacy of a request, please do not hesitate to contact a customer services agent via the SciPris portal or directly via oupsupport@scipris.com.

Thank you for your fine contribution.  On behalf of the Editors of the Oxford Open Neuroscience, we look forward to your continued contributions to the Journal.
